# Predictors and Clinical Outcomes of Long‐Term Opioid Therapy in Older Adults: A Systematic Review

**DOI:** 10.1002/phar.70151

**Published:** 2026-05-11

**Authors:** Iftekhar Ahmed, Nina E. Teo, Noha Keshk, David R. Foster

**Affiliations:** ^1^ Department of Pharmacy Practice, College of Pharmacy Purdue University West Lafayette Indiana USA; ^2^ Currently an Employee of Alnylam Pharmaceuticals, Inc. Cambridge Massachusetts USA

**Keywords:** long‐term opioid therapy, older adults, opioid, systematic review

## Abstract

**Introduction:**

Older adults have a higher prevalence of pain and are more likely to receive long‐term opioid therapy (LTOT) compared to other age groups. They are also at elevated risk of opioid‐related adverse events due to physiological changes and polypharmacy. This systematic review aimed to identify predictors and clinical outcomes associated with LTOT in older adults.

**Methods:**

We searched PubMed, Embase, and Cochrane Library to identify randomized clinical trials (RCT) and observational studies published from inception until July 31, 2025. We included studies that examined opioid use for ≥ 90 days and included participants aged ≥ 60 years. Data were synthesized using harvest plots and narrative synthesis. The Newcastle–Ottawa Scale was used for risk of bias assessment.

**Results:**

Forty‐one observational studies were included; no RCTs met the inclusion criteria. Patient‐related factors associated with LTOT across most studies were low income/wealth (*n* = 6/6 studies), depressive disorders (*n* = 6/9 studies), and dual insurance eligibility/enrollment (*n* = 4/6 studies). Prescription/dispensation‐related factors associated with LTOT across most studies were opioid use before surgery/trauma (*n* = 6/6 studies), prior/concurrent use of benzodiazepines (*n* = 6/6 studies), anxiolytics/sedatives/hypnotics (*n* = 3/4 studies), anticonvulsants (*n* = 3/4 studies), opioid use after surgery/trauma (*n* = 3/3 studies), long‐acting opioids (*n* = 3/3 studies), and longer duration of initial opioid (*n* = 2/2 studies). Regarding outcomes of LTOT, most studies reported positive associations between LTOT and hospital readmission/emergency department visit (*n* = 3/4 studies), revision surgery (*n* = 2/2 studies), health care costs (*n* = 2/2 studies), opioid overdose (*n* = 1/1 study), and falls (*n* = 1/1 study). Evidence on the association of LTOT with mortality and fractures was inconclusive.

**Conclusions:**

This systematic review identified several predictors and adverse outcomes associated with LTOT in older adults. However, no evidence exists regarding the effectiveness of LTOT for pain management in this population. Prospective studies with long‐term follow‐up are needed to address this gap and inform benefit‐harm assessment of LTOT in clinical practice.

## 1 Introduction

Pain is common among older adults. Over 50% of adults age 65 years and older in the United States report the presence of bothersome pain in the last month [1]. Similarly, the prevalence of chronic pain in the United States is 30% for people aged 65 to 84 years of age and 34% for people aged ≥ 85 years, whereas the overall prevalence in the population is 21% [2]. In addition, older adults have a higher prevalence of surgery compared to other age groups, which also contributes to higher levels of acute and chronic pain [3].

Clinical guidelines for pharmacological pain management in older adults provide varying support for commonly used medications such as acetaminophen, non‐steroidal anti‐inflammatory drugs (NSAIDs), and opioids [4]. In 2017, nearly 17% of the US population had at least one opioid prescription filled; however, this rate was significantly higher at 26% in people aged 55 to 64 years and 27% in people aged ≥ 65 years [5]. Although opioids are effective for short‐term pain management, evidence supporting benefits of long‐term opioid therapy (LTOT) is limited [6, 7]. Opioid use in older adults is particularly concerning due to increased vulnerability to adverse effects, including fractures, delirium, sedation, falls, increased pain sensitivity, immunosuppression, chronic constipation, anxiety, and depression [6, 7, 8]. These risks are compounded by the presence of comorbidities and polypharmacy [6]. Moreover, age‐related declines in hepatic and renal function may impair drug metabolism and clearance, thereby elevating the risk of opioid‐related adverse events such as overdose and respiratory depression. Nevertheless, LTOT remains prevalent in older adults, with 7% to 16% receiving opioids for ≥ 90 days [9, 10]. In addition, studies have reported positive associations between age and LTOT which means older adults are more likely to receive LTOT than younger age groups [11, 12, 13].

There is a need to better understand which patients are at risk of transitioning to LTOT and to understand the clinical outcomes associated with LTOT among older adults. Despite the prevalence of LTOT, clinical guidelines provide minimal guidance for their use in pain management in older adults due to a lack of robust evidence [6, 11, 14, 15]. Knowledge of the predictors and outcomes of LTOT will enable health care providers to more effectively evaluate the benefit‐harm balance when prescribing opioids to older adults. To address this gap in research, we performed a systematic review of published literature to answer two research questions: (i) What are the predictors of LTOT for noncancer pain in older adults? and (ii) What are the clinical outcomes of LTOT for noncancer pain in older adults?

## 2 Methods

The protocol for this systematic review was registered on PROSPERO (https://www.crd.york.ac.uk/PROSPERO/view/CRD42022334019). We reported this systematic review according to the Preferred Reporting Items for Systematic Reviews and Meta‐Analyses (PRISMA) [16]. Amendments to the original protocol have been described in Table S1. The review was performed using the COVIDENCE software tool (Veritas Health Innovation Ltd., Melbourne, Australia).

### 2.1 Search Strategy

We searched PubMed, Embase, and Cochrane Library of clinical trials to identify observational (cohort, case–control, cross‐sectional) studies and randomized clinical trials (RCT) published from inception (no start date was specified) until July 31, 2025. The search was restricted to studies published in English. The following terms were used for the literature search: (“opioid” OR “opioids”) AND (“chronic opioid” OR “chronic use” OR “chronic therapy” OR “long‐term” OR “persistent opioid” OR “persistent use” OR “prolonged opioid” OR “prolonged use” OR “continuous use” OR “continuous opioid” OR “continuous therapy”) AND (“older” OR “older adults” OR “older adult” OR “older individuals” OR “elderly” OR “elder” OR “geriatric” OR “geriatrics”).

### 2.2 Study Selection

We included prospective and retrospective cohort studies, RCTs, case–control studies, and cross‐sectional studies evaluating the predictors and/or clinical outcomes of LTOT in older adults. The definition of LTOT varies widely between studies. To remain consistent with most published studies and clinical practice [17, 18], we included studies that had examined opioid use of ≥ 90 days, defined as 90 days of continuous use, episodes lasting 90 days, or any prescription after 90 days from the index date (index dates refer to the first day of eligibility/follow‐up for noncancer pain). We excluded review articles, case series, case reports, abstract‐only studies, and qualitative studies. We included studies if the participants were older adults (minimum age for inclusion ≥ 60 years) with noncancer pain. Studies had to either exclusively include older adults or report results specific to older adults for inclusion. Studies had to have two comparison groups (participants receiving LTOT vs. participants receiving no opioid/short‐term opioids) for inclusion. We excluded studies that included patients with cancer or receiving hospice/palliative care. Two reviewers (IA and NT) independently evaluated each study against the selection criteria. Disagreements were resolved by discussion between three authors (I.A., N.T., D.F.).

### 2.3 Data Extraction

Two reviewers (I.A. and N.T.) independently extracted the following information from each study: year of publication, study design, study location, research question, study population, definition of older adult, sample size, pain conditions for opioid use, opioids prescribed/dispensed, definition of LTOT, and the incidence/prevalence of LTOT. In addition, we extracted the predictors and outcomes of LTOT based on a priori lists published in the protocol.

We created an a priori list of potential predictors of LTOT. Patient‐related predictors included demographic factors including sex, age, race, and income as well as clinical characteristics including mental health disorders, substance use disorders, pain, and other comorbidities. Prescription‐ or dispensing‐related predictors included types of opioids, long‐acting versus short‐acting opioids, number of days' supply, daily dose, and concurrent/prior use of antidepressants, benzodiazepines, gabapentin, or nonopioid analgesics. We also created an a priori list of the following potential outcomes of LTOT: (i) effectiveness of LTOT in reducing pain and its impact on quality of life; (ii) adverse outcomes of LTOT including mortality, opioid use disorder, opioid overdose, health care utilization (hospitalization, emergency department (ED) visits, revision surgery, etc.), as well as other adverse outcomes such as depressive disorder, cognitive decline, respiratory complications, falls and fractures, suicide, cardiac arrhythmia, constipation, and sleep apnea; and (iii) economic outcomes such as health care costs. If studies reported predictors or outcomes of LTOT that were not listed a priori but were clinically relevant, we included them as well.

### 2.4 Data Synthesis

Given that the studies were heterogeneous in how they defined LTOT and the comparison groups used against those receiving LTOT, we did not conduct meta‐analyses. For the predictors of LTOT, we summarized the results in harvest plots [19, 20], plotted using R Studio (Posit, Boston, Massachusetts). Harvest plots provide a graphical summary of the direction of association, strength or quality of evidence, and the number of studies supporting each conclusion. In this review, the bars in the harvest plots were grouped according to the direction of association (positive, negative, or no association). In the included studies, predictors were considered to be positively associated with LTOT if the values of the effect size and the 95% confidence interval (CI) were > 1. In contrast, predictors were considered to be negatively associated with LTOT if the values of the effect size and the 95% CI were < 1. If the 95% CI overlapped with 1 (regardless of the effect size), then there was considered to be no association. The height of the bars represents the quality of the study, which was estimated based on the risk of bias assessment scores (described below). The colors of the bars represent the pain conditions of the study participants. For ordinal variables with multiple categories (age, income, comorbidity number/score, opioid dose and duration), we created dichotomous variables by taking the two terminal categories and comparing them (higher vs. lower age, higher vs. lower opioid dose, and so on). For example, if a study reported odds ratios for three categories of age such as 65–75, 76–85, and > 85 years, we extracted odds ratios for 65–75 years (defined as lower age) and > 85 years (defined as higher age). This approach maximized comparability across studies with heterogeneous categorizations and enabled consistent pairwise comparisons for these variables across studies. The outcomes of LTOT were described narratively, given the small number of studies and large variations in how these variables were defined. For both predictors and outcomes of LTOT, we did not synthesize and report results from studies that did not perform regression analyses adjusted for covariates or did not have a clear description of the analytic approach (*n* = 2).

### 2.5 Risk of Bias (Quality) Assessment

Two reviewers (I.A. and N.K.) independently assessed the quality of evidence using the Newcastle–Ottawa Scale for observational studies. Three versions of the Newcastle‐Ottawa Scale (versions for cohort studies, case–control studies, and cross‐sectional studies) were used [21, 22]. Disagreements were resolved by consensus. We calculated a total score for each study and expressed it as a percentage by dividing the total obtained score by the maximum possible score. Studies scoring > 80% were classified as ‘good’ quality, those scoring 60%–80% as ‘fair’ quality, and those scoring < 60% as ‘poor’ quality.

## 3 Results

**FIGURE 1:**
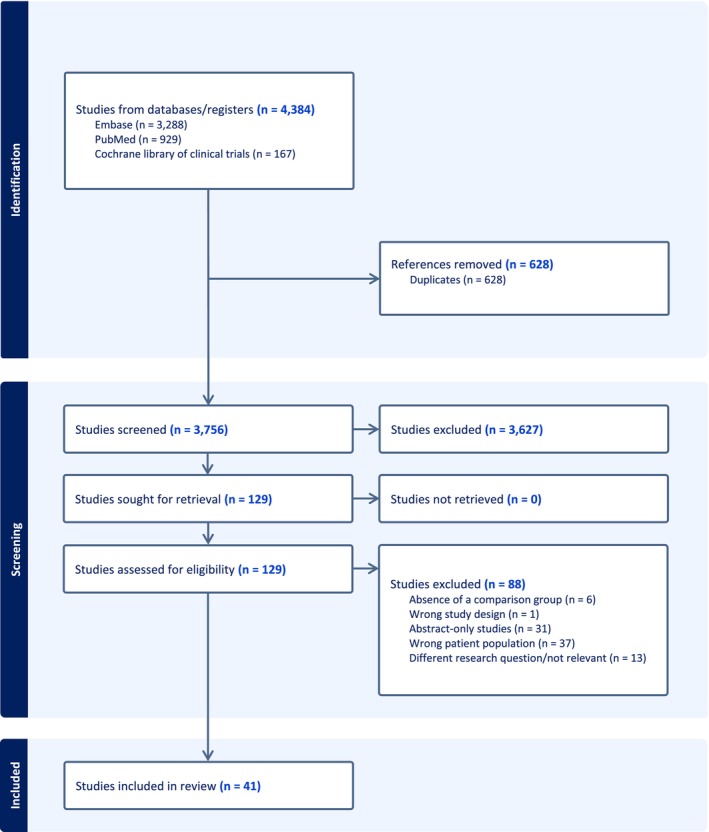
Preferred Reporting Items for Systematic Reviews and Meta‐Analyses (PRISMA) flow diagram of the study selection process.

The literature search identified 4384 studies, and 3756 of them were subjected to title and abstract screening after removing 628 duplicates. Of the 3756 studies, 129 passed title and abstract screening and underwent full‐text review, 41 of which met the inclusion criteria (Figure 1) [9, 10, 23, 24, 25, 26, 27, 28, 29, 30, 31, 32, 33, 34, 35, 36, 37, 38, 39, 40, 41, 42, 43, 44, 45, 46, 47, 48, 49, 50, 51, 52, 53, 54, 55, 56, 57, 58, 59, 60, 61]. Studies that included adults ≥ 60 years old as well as adults < 60 years old were only included if they reported data separately for the subjects ≥ 60 years old. Some studies might appear to meet the inclusion criteria due to the mean/median age of participants being ≥ 60 years. However, they were excluded since they also included participants < 60 years and did not perform any subgroup analysis for participants ≥ 60 years, making it impossible to draw conclusions specifically for older adults [62, 63].

### 3.1 Study Characteristics

Summaries and descriptions of the studies are shown in Tables S2 and S3, respectively. All 41 studies were observational, with retrospective cohort design being the most common (*n* = 34). No RCTs met the inclusion criteria. Thirty studies investigated the predictors of LTOT, eight investigated the outcomes of LTOT, and three investigated both. Most studies included patients undergoing surgery/trauma (*n* = 27). The minimum age for inclusion varied, with 65 years being the most used criterion (*n* = 30). The most frequently used definitions of LTOT were duration ≥ 90 days (*n* = 11), the last prescription/dispensation occurring after ≥ 90 days from the index date (*n* = 6), and duration ≥ 180 days (*n* = 5). Information regarding daily dose was reported in 17 studies, of which 12 reported average daily dose and four reported cumulative doses. All studies defined opioid dose as oral morphine milligram equivalents (MMEs). With the exception of one study that evaluated high‐dose chronic opioid use, LTOT was defined solely based on days' supply independent of opioid dose. Most of the studies were conducted in the United States (*n* = 27) and were published after 2021 (*n* = 24). Based on the risk of bias assessment, 23 studies were rated as ‘good’ quality, 16 as ‘fair’ quality, and two as ‘poor’ quality (Data S1).

### 3.2 Predictors of LTOT

#### 3.2.1 Patient‐Related Predictors

**FIGURE 2:**
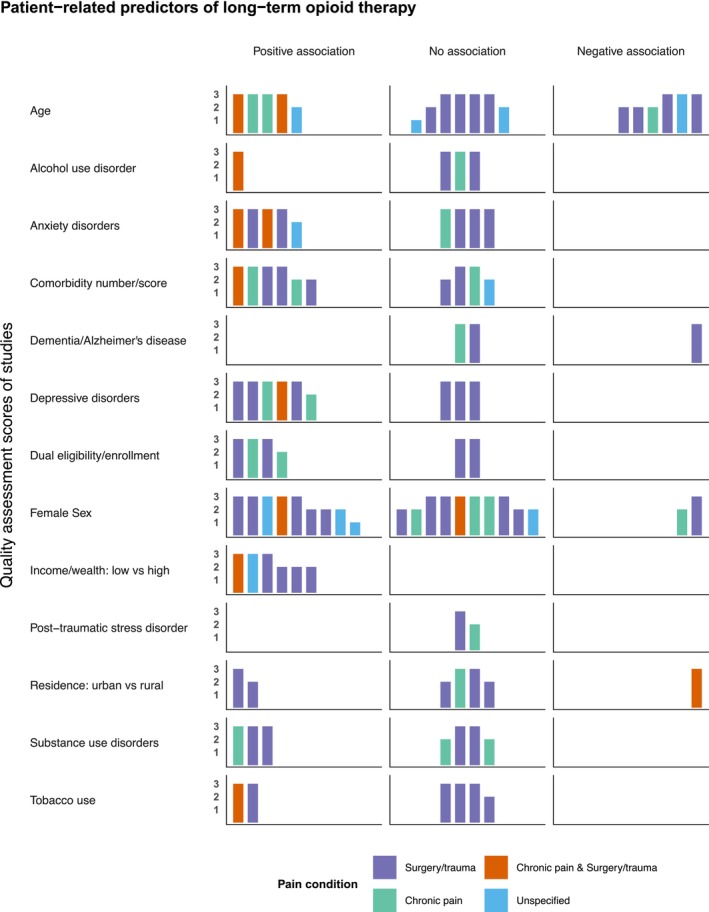
Harvest plots summarizing the evidence from the included studies (*n* = 24) on the patient‐related predictors of long‐term opioid therapy in older adults. Each study is represented by a bar and the position of the bar indicates whether the study reported positive, negative, or no association between a given risk factor and long‐term opioid therapy. The height of the bars represents the quality rating of the studies based on the Newcastle–Ottawa Scale (1 = poor, score: < 60%; 2 = fair, score: 60%–80%; 3 = good, score: > 80%). The colors of the bars represent the pain conditions for opioid use.

Figure 2 shows the harvest plots for the association between patient‐related predictors and LTOT. Patient‐related predictors were synthesized from 24 studies, 13 of which were of good quality, 10 were of fair quality, and one was of poor quality. For some predictors, most of the studies reported positive associations with LTOT. These included low household/neighborhood income/wealth (*n* = 6/6 studies, i.e., of the six studies evaluating household/neighborhood income/wealth, six found low income to have a significant positive association with LTOT), dual enrollment/eligibility in Medicare and Medicaid/Veterans Health Administration benefits (*n* = 4/6 studies), and depressive disorders (*n* = 6/9 studies). For some predictors, most of the studies found no associations. These included post−traumatic stress disorder (*n* = 2/2 studies), alcohol use disorder (*n* = 3/4 studies), and tobacco use (*n* = 4/6 studies). Factors such as age, sex, residence (urban vs. rural), comorbidity number/score, anxiety disorders, substance use disorders, and dementia/Alzheimer's disease had mixed findings. Detailed descriptions of effect sizes reported by specific studies are shown in Table 1.

**TABLE 1:** Detailed data for the patient‐related predictors of long‐term opioid therapy.

Study	Study design	Pain condition	Quality rating^a^	Predictor^b^, ^c^	Comparison group	Direction of association	Effect size estimator^d^	Effect size (95% confidence interval)^e^
Ahmed 2025 [59]	Cohort	Chronic pain and Surgery/trauma	3	Age	Received short‐term opioid therapy	Positive association	Odds ratio	1.13 (1.05–1.21)
Beyene 2023 [24]	Cohort	Chronic pain	3	Age	Received short‐term opioid therapy	Positive association	Odds ratio	1.93 (1.79–2.08)
Brown 2022 [26]	Cohort	Surgery/trauma	3	Age	Unclear	No association	Odds ratio	1 (0.99–1.01)
Chui 2018 [28]	Cross‐sectional	Chronic pain	2	Age	Received short‐term opioid therapy	Negative association	Odds ratio	0.85 (0.79–0.91)
Delaney 2020 [31]	Cohort	Surgery/trauma	3	Age	Unclear	No association	Odds ratio	1.24 (0.40–3.90)
Gopalakrishnan 2022 [33]	Cohort	Surgery/trauma	3	Age	Received short‐term opioid therapy	Negative association	Odds ratio	0.98 (0.98–0.99)
Hadlandsmyth 2024 [34]	Cohort	Surgery/trauma	2	Age	Received short‐term opioid therapy	No association	Odds ratio	0.97 (0.93–1.01)
Hereford 2022 [35]	Cohort	Surgery/trauma	2	Age	Received short‐term opioid therapy	Negative association	Odds ratio	0.96 (0.92–0.99)
Hunnicut 2018 [10]	Cross‐sectional	Unspecified	3	Age	Received short‐term and medium‐term opioid therapy + did not receive any opioid	Negative association	Prevalence ratio	0.94 (0.92–0.97)
Johnson 2024 [39]	Cohort	Surgery/trauma	2	Age	Received short‐term opioid therapy	Negative association	Odds ratio	0.56 (0.48–0.66)
Karmali 2020 [9]	Cohort	Chronic pain	3	Age	Received short‐term opioid therapy	Positive association	Odds ratio	1.64 (1.37–1.98)
Karp 2013 [40]	Cohort	Unspecified	1	Age	Both infrequent and non‐users of opioids	No association	Odds ratio	1.9 (0.71–5.09)
Ly 2019 [42]	Cross‐sectional	Surgery/trauma	3	Age	Received short‐term opioid therapy	No association	Percentage point difference in probability	0.002 (−0.05–0.06)
Moffat 2020 [58]	Cohort	Unspecified	2	Age	Received short‐term opioid therapy	Positive association	Hazard ratio	1.06 (1.06–1.07)
Musich 2019 [43]	Cohort	Chronic pain and Surgery/trauma	3	Age	Received short‐term opioid therapy	Positive association	Odds ratio	1.19 (*p* < 0.0001)
Okike 2023 [47]	Cohort	Surgery/trauma	3	Age	Did not receive any opioid	Negative association	Incident rate ratio	0.25 (0.23–0.26)
Santosa 2020 [49]	Cohort	Surgery/trauma	3	Age	Received short‐term opioid therapy	No association	Odds ratio	1.09 (0.99–1.21)
Tevik 2021 [56]	Cohort	Unspecified	2	Age	Received short‐term opioid therapy	No association	Odds ratio	0.97 (0.92–1.03)
Ahmed 2025 [59]	Cohort	Chronic pain & Surgery/trauma	3	Alcohol use disorder	Received short‐term opioid therapy	Positive association	Odds ratio	1.38 (1.23–1.54)
Beyene 2023 [24]	Cohort	Chronic pain	3	Alcohol use disorder	Received short‐term opioid therapy	No association	Odds ratio	1.13 (0.89–1.42)
Daoust 2018 [30]	Cohort	Surgery/trauma	3	Alcohol use disorder	Did not receive any opioid	No association	Odds ratio	1.28 (0.94–1.74)
Okike 2023 [47]	Cohort	Surgery/trauma	3	Alcohol use disorder	Did not receive any opioid	No association	Incident rate ratio	1.43 (0.79–2.61)
Ahmed 2025 [59]	Cohort	Chronic pain & Surgery/trauma	3	Anxiety disorders	Received short‐term opioid therapy	Positive association	Odds ratio	1.36 (1.15–1.60) 1.45 (1.22–1.71) 1.50 (1.01–2.19)
Brown 2022 [26]	Cohort	Surgery/trauma	3	Anxiety disorders	Unclear	Positive association	Odds ratio	1.17 (1.02–1.34)
Daoust 2018 [30]	Cohort	Surgery/trauma	3	Anxiety disorders	Did not receive any opioid	No association	Odds ratio	1.12 (0.99–1.27)
Delaney 2020 [31]	Cohort	Surgery/trauma	3	Anxiety disorders	Unclear	No association	Odds ratio	1.36 (0.49–3.81)
Karmali 2020 [9]	Cohort	Chronic pain	3	Anxiety disorders	Received short‐term opioid therapy	No association	Odds ratio	1.41 (1.00–1.98)
Moffat 2020 [58]	Cohort	Unspecified	2	Anxiety disorders	Received short‐term opioid therapy	Positive association	Hazard ratio	1.3 (1.15–1.47)
Musich 2019 [43]	Cohort	Chronic pain & Surgery/trauma	3	Anxiety disorders	Received short‐term opioid therapy	Positive association	Odds ratio	1.26 (*p* < 0.0001)
Okike 2023 [47]	Cohort	Surgery/trauma	3	Anxiety disorders	Did not receive any opioid	Positive association	Incident rate ratio	1.43 (1.01–2.02)
Santosa 2020 [49]	Cohort	Surgery/trauma	3	Anxiety disorders	Received short‐term opioid therapy	No association	Odds ratio	1.07 (1.00–1.15)
Ahmed 2025 [59]	Cohort	Chronic pain & Surgery/trauma	3	Comorbidity number/score	Received short‐term opioid therapy	Positive association	Odds ratio	1.55 (1.45–1.65)
Beyene 2023 [24]	Cohort	Chronic pain	3	Comorbidity number/score	Received short‐term opioid therapy	Positive association	Odds ratio	2.09 (1.78–2.46)
Brown 2022 [26]	Cohort	Surgery/trauma	3	Comorbidity number/score	Unclear	Positive association	Odds ratio	2.09 (1.66–2.64)
Chui 2018 [28]	Cross‐sectional	Chronic pain	2	Comorbidity number/score	Received short‐term opioid therapy	Positive association	Odds ratio	1.08 (1.01–1.15)
Delaney 2020 [31]	Cohort	Surgery/trauma	3	Comorbidity number/score	Unclear	No association	Odds ratio	1.13 (0.97–1.33)
Hadlandsmyth 2024 [34]	Cohort	Surgery/trauma	2	Comorbidity number/score	Received short‐term opioid therapy	No association	Odds ratio	1.03 (0.96–1.10)
Johnson 2021 [38]	Cohort	Surgery/trauma	2	Comorbidity number/score	Received short‐term opioid therapy	Positive association	Odds ratio	1.99 (1.72–2.30) 1.46 (1.31–1.64)
Karmali 2020 [9]	Cohort	Chronic pain	3	Comorbidity number/score	Received short‐term opioid therapy	No association	Odds ratio	1.02 (0.98–1.06)
Moffat 2020 [58]	Cohort	Unspecified	2	Comorbidity number/score	Received short‐term opioid therapy	No association	Hazard ratio	1.02 (0.99–1.04)
Santosa, 2020 [49]	Cohort	Surgery/trauma	3	Comorbidity number/score	Received short‐term opioid therapy	Positive association	Odds ratio	1.71 (1.58–1.84)
Beyene 2023 [24]	Cohort	Chronic pain	3	Dementia/Alzheimer's disease	Received short‐term opioid therapy	No association	Odds ratio	1.04 (0.9–1.2)
Ly 2019 [42]	Cross‐sectional	Surgery/trauma	3	Dementia/Alzheimer's disease	Received short‐term opioid therapy	No association	Percentage point difference in probability	−1.07 (−2.47–0.34)
Okike 2023 [47]	Cohort	Surgery/trauma	3	Dementia/Alzheimer's disease	Did not receive any opioid	Negative association	Incident rate ratio	0.54 (0.41–0.72)
Brown 2022 [26]	Cohort	Surgery/trauma	3	Depressive disorders	Unclear	Positive association	Odds ratio	1.15 (1.01–1.31)
Chui 2018 [28]	Cross‐sectional	Chronic pain	2	Depressive disorders	Received short‐term opioid therapy	Positive association	Odds ratio	1.31 (1.14–1.51)
Daoust 2018 [30]	Cohort	Surgery/trauma	3	Depressive disorders	Did not receive any opioid	Positive association	Odds ratio	1.32 (1.13–1.53)
Delaney 2020 [31]	Cohort	Surgery/trauma	3	Depressive disorders	Unclear	No association	Odds ratio	1.25 (0.40–3.97)
Karmali 2020 [9]	Cohort	Chronic pain	3	Depressive disorders	Received short‐term opioid therapy	Positive association	Odds ratio	1.29 (1.04–1.61)
Ly 2019 [42]	Cross‐sectional	Surgery/trauma	3	Depressive disorders	Received short‐term opioid therapy	No association	Percentage point difference in probability	0.35 (−0.34–1.04)
Musich, 2019 [43]	Cohort	Chronic pain and Surgery/trauma	3	Depressive disorders	Received short‐term opioid therapy	Positive association	Odds ratio	1.38 (*p* < 0.0001)
Okike 2023 [47]	Cohort	Surgery/trauma	3	Depressive disorders	Did not receive any opioid	No association	Incident rate ratio	1.08 (0.69–1.71)
Santosa 2020 [49]	Cohort	Surgery/trauma	3	Depressive disorders	Received short‐term opioid therapy	Positive association	Odds ratio	1.16 (1.09–1.24)
Brown 2022 [26]	Cohort	Surgery/trauma	3	Dual enrollment in Medicare and Medicaid/VHA benefits	Unclear	Positive association	Odds ratio	1.29 (1.20–1.39)
Chui 2018 [28]	Cross‐sectional	Chronic pain	2	Dual enrollment in Medicare and Medicaid/VHA benefits	Received short‐term opioid therapy	Positive association	Odds ratio	4.61 (4.05–5.25)
Delaney 2020 [31]	Cohort	Surgery/trauma	3	Dual enrollment in Medicare and Medicaid/VHA benefits	Unclear	No association	Odds ratio	1.33 (0.43–4.20)
Karmali 2020 [9]	Cohort	Chronic pain	3	Dual enrollment in Medicare and Medicaid/VHA benefits	Received short‐term opioid therapy	Positive association	Odds ratio	1.83 (1.6–2.1)
Ly 2019 [42]	Cross‐sectional	Surgery/trauma	3	Dual enrollment in Medicare and Medicaid/VHA benefits	Received short‐term opioid therapy	No association	Percentage point difference in probability	1.47 (−0.83 to 3.77)
Santosa 2020 [49]	Cohort	Surgery/trauma	3	Dual enrollment in Medicare and Medicaid/VHA benefits	Received short‐term opioid therapy	Positive association	Odds ratio	1.45 (1.35–1.55)
Ahmed 2025 [59]	Cohort	Chronic pain & Surgery/trauma	3	Female Sex	Received short‐term opioid therapy	No association	Odds ratio	1.05 (1.00–1.10)
Beyene 2023 [24]	Cohort	Chronic pain	3	Female Sex	Received short‐term opioid therapy	No association	Odds ratio	1.03 (0.97–1.09)
Brown 2022 [26]	Cohort	Surgery/trauma	3	Female Sex	Unclear	Positive association	Odds ratio	1.2 (1.09–1.32)
Chui 2018 [28]	Cross‐sectional	Chronic pain	2	Female Sex	Received short‐term opioid therapy	No association	Odds ratio	0.9 (0.71–1.15)
Daoust 2018 [30]	Cohort	Surgery/trauma	3	Female Sex	Did not receive any opioid	Positive association	Odds ratio	1.27 (1.16–1.38)
Delaney 2020 [31]	Cohort	Surgery/trauma	3	Female Sex	Unclear	No association	Odds ratio	1.10 (0.71–1.72)
Gopalakrishnan 2022 [33]	Cohort	Surgery/trauma	3	Female Sex	Received short‐term opioid therapy	Negative association	Odds ratio	0.83 (0.79–0.88)
Hadlandsmyth 2024 [34]	Cohort	Surgery/trauma	2	Female Sex	Received short‐term opioid therapy	No association	Odds ratio	1.33 (0.73–2.43)
Hereford 2022 [35]	Cohort	Surgery/trauma	2	Female Sex	Received short‐term opioid therapy	No association	Odds ratio	0.57 (0.28–1.16)
Hunnicut 2018 [10]	Cross‐sectional	Unspecified	3	Female Sex	Received short‐term and medium‐term opioid therapy + did not receive any opioid	Positive association	Prevalence ratio	1.21 (1.18–1.23)
Johnson 2021 [38]	Cohort	Surgery/trauma	2	Female Sex	Received short‐term opioid therapy	Positive association	Odds ratio	1.33 (1.27–1.40) 1.26 (1.22–1.3)
Johnson 2024 [39]	Cohort	Surgery/trauma	2	Female Sex	Received short‐term opioid therapy	Positive association	Odds ratio	1.08 (1.01–1.16)
Karmali 2020 [9]	Cohort	Chronic pain	3	Female Sex	Received short‐term opioid therapy	No association	Odds ratio	0.99 (0.87–1.12)
Karp 2013 [40]	Cohort	Unspecified	1	Female Sex	Both infrequent and non‐users of opioids	Positive association	Odds ratio	4.06 (1.98–8.35)
Ly 2019 [42]	Cross‐sectional	Surgery/trauma	3	Female Sex	Received short‐term opioid therapy	No association	Percentage point difference in probability	−0.17 (−0.64–0.29)
Moffat 2020 [58]	Cohort	Unspecified	2	Female Sex	Received short‐term opioid therapy	Positive association	Hazard ratio	1.44 (1.26–1.65)
Musich 2019 [43]	Cohort	Chronic pain and Surgery/trauma	3	Female Sex	Received short‐term opioid therapy	Positive association	Odds ratio	1.19 (*p* < 0.0001)
Oh 2019 [46]	Cohort	Chronic pain	2	Female Sex	Received short‐term opioid therapy	Negative association	Odds ratio	0.72 (0.52–0.98)
Okike 2023 [47]	Cohort	Surgery/trauma	3	Female Sex	Did not receive any opioid	Positive association	Incident rate ratio	1.47 (1.12–1.91)
Santosa 2020 [49]	Cohort	Surgery/trauma	3	Female Sex	Received short‐term opioid therapy	No association	Odds ratio	1.02 (0.97–1.07)
Tevik 2021 [56]	Cohort	Unspecified	2	Female Sex	Received short‐term opioid therapy	No association	Odds ratio	1.32 (0.63–2.78)
Johnson 2021 [38]	Cohort	Surgery/trauma	2	Income/wealth: low vs. high	Received short‐term opioid therapy	Positive association	Odds ratio	1.41 (1.32–1.52) 1.43 (1.35–1.49)
Johnson 2024 [39]	Cohort	Surgery/trauma	2	Income/wealth: low vs. high	Received short‐term opioid therapy	Positive association	Odds ratio	1.24 (1.11–1.38)
Musich 2019 [43]	Cohort	Chronic pain & Surgery/trauma	3	Income/wealth: low vs. high	Received short‐term opioid therapy	Positive association	Odds ratio	1.49 (*p* < 0.0001)
Nestvolt 2024 [44]	Case–control	Unspecified	3	Income/wealth: low vs. high	Received short‐term opioid therapy	Positive association	Odds ratio	1.23 (1.19–1.27)
Okike 2023 [47]	Cohort	Surgery/trauma	3	Income/wealth: low vs. high	Did not receive any opioid	Positive association	Incident rate ratio	3.28 (2.43–4.30)
Risbo 2025 [60]	Cohort	Surgery/trauma	2	Income/wealth: low vs. high	Unclear	Positive association	Risk ratio	1.28 (1.23–1.34)
Chui 2018 [28]	Cross‐sectional	Chronic pain	2	Post‐traumatic stress disorder	Received short‐term opioid therapy	No association	Odds ratio	1.05 (0.93–1.19)
Okike 2023 [47]	Cohort	Surgery/trauma	3	Post‐traumatic stress disorder	Did not receive any opioid	No association	Incident rate ratio	1.14 (0.14–9.49)
Brown 2022 [26]	Cohort	Surgery/trauma	3	Residence: urban vs. rural	Unclear	Positive association	Odds ratio	1.19 (1.06–1.32)
Hadlandsmyth 2024 [34]	Cohort	Surgery/trauma	2	Residence: urban vs. rural	Received short‐term opioid therapy	No association	Odds ratio	1.27 (0.99–1.61)
Johnson 2021 [38]	Cohort	Surgery/trauma	2	Residence: urban vs. rural	Received short‐term opioid therapy	Positive association	Odds ratio	1.12 (1.05–1.20) 1.14 (1.09–1.20)
Johnson 2024 [39]	Cohort	Surgery/trauma	2	Residence: urban vs. rural	Received short‐term opioid therapy	No association	Odds ratio	0.9 (0.82–1.00)
Karmali 2020 [9]	Cohort	Chronic pain	3	Residence: urban vs. rural	Received short‐term opioid therapy	No association	Odds ratio	0.97 (0.82–1.14)
Musich, 2019 [43]	Cohort	Chronic pain and Surgery/trauma	3	Residence: urban vs. rural	Received short‐term opioid therapy	Negative association	Odds ratio	0.9 (*p* = 0.0001)
Santosa, 2020 [49]	Cohort	Surgery/trauma	3	Residence: urban vs. rural	Received short‐term opioid therapy	No association	Odds ratio	1.02 (0.96–1.08)
Beyene 2023 [24]	Cohort	Chronic pain	3	Substance use disorders	Received short‐term opioid therapy	Positive association	Odds ratio	1.52 (1.35–7.72)
Brown 2022 [26]	Cohort	Surgery/trauma	3	Substance use disorders	Unclear	No association	Odds ratio	1.13 (0.93–1.38)
Chui 2018 [28]	Cross‐sectional	Chronic pain	2	Substance use disorders	Received short‐term opioid therapy	No association	Odds ratio	1.11 (0.99–1.25)
Gopalakrishnan 2022 [33]	Cohort	Surgery/trauma	3	Substance use disorders	Received short‐term opioid therapy	Positive association	Odds ratio	1.14 (1.06–1.22)
Okike 2023 [47]	Cohort	Surgery/trauma	3	Substance use disorders	Did not receive any opioid	No association	Incident rate ratio	1.37 (0.85–2.21)
Santosa 2020 [49]	Cohort	Surgery/trauma	3	Substance use disorders	Received short‐term opioid therapy	Positive association	Odds ratio	1.38 (1.2–1.59)
Solomon 2006 [53]	Cohort	Chronic pain	2	Substance use disorders	Unclear	No association	Odds ratio	0.8 (0.2–3.3) 2.7 (0.9–8.7) 0.5 (0.1–3.8)
Ahmed 2025 [59]	Cohort	Chronic pain & Surgery/trauma	3	Tobacco use	Received short‐term opioid therapy	Positive association	Odds ratio	1.31 (1.23–1.40)
Brown 2022 [26]	Cohort	Surgery/trauma	3	Tobacco use	Unclear	No association	Odds ratio	0.99 (0.89–1.10)
Delaney 2020 [31]	Cohort	Surgery/trauma	3	Tobacco use	Unclear	No association	Odds ratio	0.88 (0.40–1.92)
Hereford 2022 [35]	Cohort	Surgery/trauma	2	Tobacco use	Received short‐term opioid therapy	No association	Odds ratio	0.46 (0.15–1.38)
Okike 2023 [47]	Cohort	Surgery/trauma	3	Tobacco use	Did not receive any opioid	Positive association	Incident rate ratio	1.4 (1.09–1.79)
Santosa 2020 [49]	Cohort	Surgery/trauma	3	Tobacco use	Received short‐term opioid therapy	No association	Odds ratio	1.03 (0.97–1.09)

Abbreviations: LTOT, long‐term opioid therapy; VHA, veterans health administration.

^a^ Quality rating is based on the Newcastle‐Ottawa Scale; 1 = poor, score: < 60%; 2 = fair, score: 60%–80%; 3 = good, score: > 80%.

^b^ Some variables (age, income, comorbidity number/score) were modeled as either categorical or continuous variables in the original studies.

^c^ For ordinal variables with multiple categories (age, income, comorbidity number/score), only the two terminal categories were compared.

^d^ All estimates were adjusted for covariates.

^e^ The effect sizes and their confidence intervals were inverted (1/effect size and 1/confidence interval) for some studies to ensure consistent reference groups for variables across all studies. One study (Musich [48]) reported *p*‐values instead of confidence intervals. Three studies (Solomon [53], Johnson [38], Ahmed [59]) reported odds ratios for specific subgroups rather than the full sample for certain variables, resulting in multiple odds ratios per study.

#### 3.2.2 Prescription/Dispensation‐Related Predictors

**FIGURE 3:**
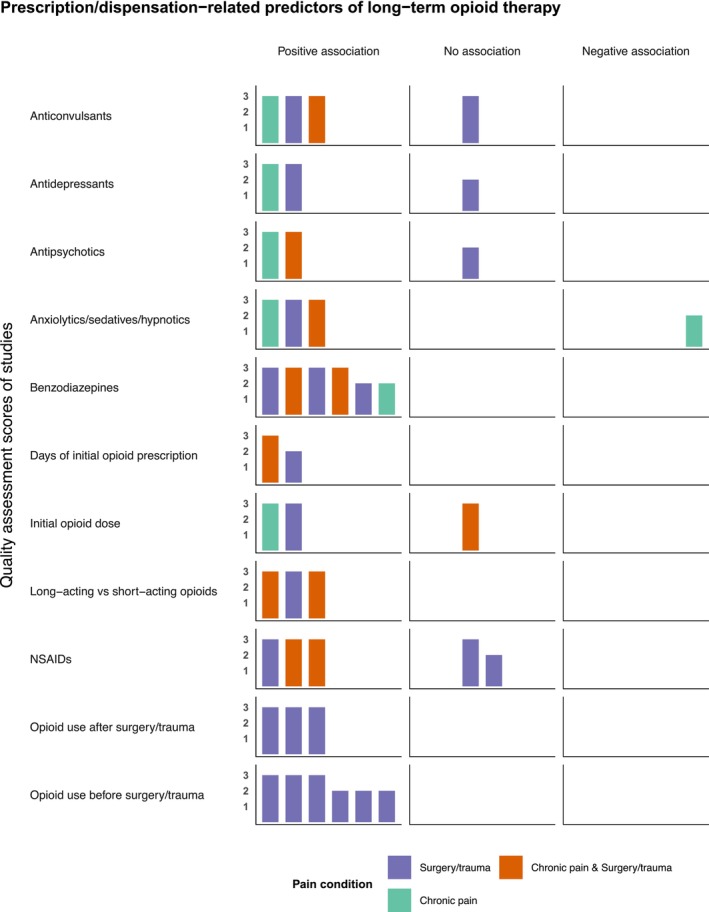
Harvest plots summarizing the evidence from the included studies (*n* = 16) on the prescription/dispensation‐related predictors of long‐term opioid therapy in older adults. Each study is represented by a bar and the position of the bar indicates whether the study reported positive, negative, or no association between a given risk factor and long‐term opioid therapy. The height of the bars represents the quality rating of the studies based on the Newcastle–Ottawa Scale (1 = poor, score: < 60%; 2 = fair, score: 60%–80%; 3 = good, score: > 80%). The colors of the bars represent the pain conditions for opioid use. NSAIDs, nonsteroidal anti‐inflammatory drugs.

Figure 3 shows the harvest plots for the association between prescription/dispensation‐related predictors and LTOT. Prescription/dispensation‐related predictors were synthesized from 16 studies, 10 of which were of good quality and six were of fair quality. Factors positively associated with LTOT included opioid use before surgery/trauma (*n* = 6/6 studies), opioid use after surgery/trauma (*n* = 3/3 studies), use of long‐acting opioids (*n* = 3/3 studies), and days' supply of initial opioid prescription (*n* = 2/2 studies). Most studies investigating the association between nonopioid concurrent/prior medications and LTOT identified concurrent/prior use of benzodiazepines (*n* = 6/6 studies), anxiolytics/sedatives/hypnotics (*n* = 3/4 studies), and anticonvulsants (*n* = 3/4 studies) as risk factors for LTOT. Evidence for initial opioid dose, concurrent/prior use of antidepressants, antipsychotics, and NSAIDs was mixed. Detailed descriptions of effect sizes reported by specific studies are found in Table 2.

**TABLE 2:** Detailed data for the prescription/dispensation‐related predictors of long‐term opioid therapy.

Study	Study design	Pain condition	Quality rating^a^	Predictor^b^, ^c^	Comparison group	Direction of association	Effect size estimator^d^	Effect size (95% confidence interval)^e^
Ahmed 2025 [59]	Cohort	Chronic pain & Surgery/trauma	3	Anticonvulsants	Received short‐term opioid therapy	Positive association	Odds ratio	1.57 (1.47–1.67)
Beyene 2023 [24]	Cohort	Chronic pain	3	Anticonvulsants	Received short‐term opioid therapy	Positive association	Odds ratio	2.07 (1.89–2.26)
Chen 2022 [27]	Cohort	Surgery/trauma	3	Anticonvulsants	Received short‐term opioid therapy	No association	Relative risk	1.07 (0.91–1.26)
Gopalakrishnan 2022 [33]	Cohort	Surgery/trauma	3	Anticonvulsants	Received short‐term opioid therapy	Positive association	Odds ratio	1.25 (1.17–1.33)
Beyene 2023 [24]	Cohort	Chronic pain	3	Antidepressants	Received short‐term opioid therapy	Positive association	Odds ratio	1.5 (1.41–1.59)
Gopalakrishnan 2022 [33]	Cohort	Surgery/trauma	3	Antidepressants	Received short‐term opioid therapy	Positive association	Odds ratio	1.16 (1.10–1.23)
Johnson 2024 [39]	Cohort	Surgery/trauma	2	Antidepressants	Received short‐term opioid therapy	No association	Odds ratio	1.09 (0.97–1.22)
Beyene 2023 [24]	Cohort	Chronic pain	3	Antipsychotics	Received short‐term opioid therapy	Positive association	Odds ratio	1.96 (1.78–2.17)
Johnson 2024 [39]	Cohort	Surgery/trauma	2	Antipsychotics	Received short‐term opioid therapy	No association	Odds ratio	1.09 (0.84–1.43)
Musich 2019 [43]	Cohort	Chronic pain & Surgery/trauma	3	Antipsychotics	Received short‐term opioid therapy	Positive association	Odds ratio	1.43 (*p* < 0.0001)
Beyene 2023 [24]	Cohort	Chronic pain	3	Anxiolytics/sedatives/hypnotics	Received short‐term opioid therapy	Positive association	Odds ratio	1.31 (1.23–1.39)
Gopalakrishnan 2022 [33]	Cohort	Surgery/trauma	3	Anxiolytics/sedatives/hypnotics	Received short‐term opioid therapy	Positive association	Odds ratio	1.27 (1.19–1.36)
Musich 2019 [43]	Cohort	Chronic pain & Surgery/trauma	3	Anxiolytics/sedatives/hypnotics	Received short‐term opioid therapy	Positive association	Odds ratio	1.79 (*p* < 0.0001)
Oh 2019 [46]	Cohort	Chronic pain	2	Anxiolytics/sedatives/hypnotics	Received short‐term opioid therapy	Negative association	Odds ratio	0.57 (0.39–0.83)
Ahmed 2025 [59]	Cohort	Chronic pain & Surgery/trauma	3	Benzodiazepines	Received short‐term opioid therapy	Positive association	Odds ratio	1.16 (1.08–1.25)
Gopalakrishnan 2022 [33]	Cohort	Surgery/trauma	3	Benzodiazepines	Received short‐term opioid therapy	Positive association	Odds ratio	1.44 (1.32–1.57)
Johnson 2024 [39]	Cohort	Surgery/trauma	2	Benzodiazepines	Received short‐term opioid therapy	Positive association	Odds ratio	1.45 (1.33–1.58)
Musich 2019 [43]	Cohort	Chronic pain & Surgery/trauma	3	Benzodiazepines	Received short‐term opioid therapy	Positive association	Odds ratio	1.26 (*p* < 0.0001)
Santosa 2020 [49]	Cohort	Surgery/trauma	3	Benzodiazepines	Received short‐term opioid therapy	Positive association	Odds ratio	4.83 (4.08–5.71)
Solomon 2006 [53]	Cohort	Chronic pain	2	Benzodiazepines	Unclear	Positive association	Odds ratio	1.5 (1.2–1.8) 1.8 (1.5–2.2) 1.9 (1.5–2.6)
Ahmed 2025 [59]	Cohort	Chronic pain & Surgery/trauma	3	Days of initial opioid prescription	Received short‐term opioid therapy	Positive association	Odds ratio	11.52 (8.61–15.66) 15.97 (11.68–22.28) 14.49 (8.65–24.51)
Hadlandsmyth 2024 [34]	Cohort	Surgery/trauma	2	Days of initial opioid prescription	Received short‐term opioid therapy	Positive association	Odds ratio	1.07 (1.06–1.08)
Ahmed 2025 [59]	Cohort	Chronic pain & Surgery/trauma	3	Initial opioid dose	Received short‐term opioid therapy	No association	Odds ratio	1.11 (0.79–1.53) 1.04 (0.74–1.45) 0.65 (0.31–1.31)
Beyene 2023 [24]	Cohort	Chronic pain	3	Initial opioid dose	Received short‐term opioid therapy	Positive association	Odds ratio	1.78 (1.61–1.98)
Santosa 2020 [49]	Cohort	Surgery/trauma	3	Initial opioid dose	Received short‐term opioid therapy	Positive association	Odds ratio	1.22 (1.09–1.36)
Ahmed 2025 [59]	Cohort	Chronic pain & Surgery/trauma	3	Long‐acting vs. short‐acting opioids	Received short‐term opioid therapy	Positive association	Odds ratio	1.72 (1.21–2.44)
Musich 2019 [43]	Cohort	Chronic pain & Surgery/trauma	3	Long‐acting vs. short‐acting opioids	Received short‐term opioid therapy	Positive association	Odds ratio	26.24 (p < 0.0001)
Santosa 2020 [49]	Cohort	Surgery/trauma	3	Long‐acting vs. short‐acting opioids	Received short‐term opioid therapy	Positive association	Odds ratio	2.87 (2.18–3.76)
Ahmed 2025 [59]	Cohort	Chronic pain & Surgery/trauma	3	NSAIDs	Received short‐term opioid therapy	Positive association	Odds ratio	1.24 (1.17–1.31)
Gopalakrishnan 2022 [33]	Cohort	Surgery/trauma	3	NSAIDs	Received short‐term opioid therapy	Positive association	Odds ratio	1.21 (1.15–1.27)
Johnson 2024 [39]	Cohort	Surgery/trauma	2	NSAIDs	Received short‐term opioid therapy	No association	Odds ratio	1.05 (0.96–1.14)
Musich 2019 [43]	Cohort	Chronic pain & Surgery/trauma	3	NSAIDs	Received short‐term opioid therapy	Positive association	Odds ratio	1.62 (*p* < 0.0001)
Okike 2023 [47]	Cohort	Surgery/trauma	3	NSAIDs	Did not receive any opioid	No association	Incident rate ratio	1.2 (0.82–1.78)
Alam 2012 [23]	Cohort	Surgery/trauma	3	Opioid use after surgery/trauma	Did not receive any opioid	Positive association	Odds ratio	1.44 (1.39–1.50)
Brown 2022 [26]	Cohort	Surgery/trauma	3	Opioid use after surgery/trauma	Unclear	Positive association	Odds ratio	2.26 (2.00–2.56)
Daoust 2018 [30]	Cohort	Surgery/trauma	3	Opioid use after surgery/trauma	Did not receive any opioid	Positive association	Odds ratio	3.05 (2.83–3.29)
Brown 2022 [26]	Cohort	Surgery/trauma	3	Opioid use before surgery/trauma	Unclear	Positive association	Odds ratio	4.3 (3.74–4.95)
Daoust 2018 [30]	Cohort	Surgery/trauma	3	Opioid use before surgery/trauma	Did not receive any opioid	Positive association	Odds ratio	11.4 (10.5–12.5)
Hereford 2022 [35]	Cohort	Surgery/trauma	2	Opioid use before surgery/trauma	Received short‐term opioid therapy	Positive association	Odds ratio	3.3 (1.4–7.7)
Johnson 2024 [39]	Cohort	Surgery/trauma	2	Opioid use before surgery/trauma	Received short‐term opioid therapy	Positive association	Odds ratio	4.47 (4.16–4.79)
Nørgård 2024 [45]	Cohort	Surgery/trauma	2	Opioid use before surgery/trauma	Unclear	Positive association	Odds ratio	10.37 (6.77–15.88)
Santosa 2020 [49]	Cohort	Surgery/trauma	3	Opioid use before surgery/trauma	Received short‐term opioid therapy	Positive association	Odds ratio	1.67 (1.58–1.77)

Abbreviations: LTOT, long‐term opioid therapy; NSAIDs, nonsteroidal anti‐inflammatory drugs.

^a^ Quality rating is based on the Newcastle‐Ottawa Scale; 1 = poor, score: < 60%; 2 = fair, score: 60%–80%; 3 = good, score: > 80%.

^b^ Some variables (initial opioid dose, days of initial opioid prescription) were modeled as either categorical or continuous variables in the original studies.

^c^ For ordinal variables with multiple categories (initial opioid dose, days of initial opioid prescription), only the two terminal categories were compared.

^d^ All estimates were adjusted for covariates.

^e^ The effect sizes and their confidence intervals were inverted (1/effect size and 1/confidence interval) for some studies to ensure consistent reference groups for variables across all studies. One study (Musich [43]) reported *p*‐values instead of confidence intervals. Two studies (Solomon [53], Ahmed [59]) reported odds ratios for specific subgroups rather than the full sample for certain variables, resulting in multiple odds ratios per study.

### 3.3 Outcomes of LTOT

None of the included studies investigating outcomes of LTOT assessed the effectiveness of LTOT in improving pain. The studies primarily assessed adverse outcomes of LTOT (Table 3).

**TABLE 3:** Clinical outcomes of long‐term opioid therapy in older adults.

Outcome category	Study	Study quality based on risk of bias assessment	Outcome variable	Predictor variable	Effect size (95% confidence interval)
Mortality	Santosa 2023 [50]	Good	Mortality (unspecified) within the 6 to 12 months after surgery	LTOT vs. short‐term opioid use and no opioid use combined after surgery	AHR: 3.44 (2.99–3.96)
	Kim 2019 [41]	Good	All‐cause mortality within 90 days after surgery	LTOT vs. no opioid use before surgery	AHR: 1.00 (0.71–1.40)
Opioid overdose	Kim 2019 [41]	Good	Opioid overdose within 90 days after surgery	LTOT vs. no opioid use before surgery	AHR: 9.60 (3.16–29.15)
Hospital readmission/ED visit	Santosa 2023 [50]	Good	Hospital readmission/ED visit related to a respiratory event within the 6 to 12 months after surgery	LTOT vs. short‐term opioid use and no opioid use combined after surgery	AOR: 1.67 (1.49–1.86)
			Hospital readmission/ED visit for a pain or opioid event within the 6 to 12 months after surgery	LTOT vs. short‐term opioid use and no opioid use combined after surgery	AOR: 1.68 (1.55–1.82)
	Kim 2019 [41]	Good	Hospital readmission within 90 days after surgery	LTOT vs. no opioid use before surgery	AHR: 1.17 (1.10–1.26)
	Ravi 2021 [48]	Good	Hospital readmission/ED visit within 30 days after surgery	LTOT vs. no opioid use before surgery	AHR: 1.07 (1.00–1.14)
	Tang 2022 [55]	Good	Opioid‐related hospital readmission within 30 days after discharge	LTOT vs. no opioid use before surgery	AOR: 3.70 (2.71–5.04)
			Pain‐related hospital readmission within 30 days after discharge	LTOT vs. no opioid use before surgery	AOR: 1.62 (1.53–1.71)
			Respiratory‐related hospital readmission within 30 days after discharge	LTOT vs. no opioid use before surgery	AOR: 1.44 (1.34–1.55)
			All‐cause hospital readmission within 30 days after discharge	LTOT vs. no opioid use before surgery	AOR: 1.23 (1.18–1.29)
Revision surgery	Kim 2019 [41]	Good	Revision surgery within 90 days after surgery	LTOT vs. no opioid use before surgery	AHR: 1.49 (1.12–1.98)
	Ravi 2021 [48]	Good	Revision surgery within 1 year after surgery	LTOT vs. no opioid use before surgery	AHR: 1.35 (1.01–1.80)
Health care cost	Johnson 2024 [39]	Fair	Health care cost during first postoperative year	LTOT vs. short‐term opioid use after surgery	AMR: 1.44 (1.37–1.51)
	Johnson 2022 [37]	Fair	1‐year postoperative costs	LTOT vs. short‐term opioid use after surgery	AMR in hip surgery cohort: 1.43 (1.42–1.45) AMR in knee surgery cohort: 1.41 (1.40–1.42)
Falls/fractures	Santosa 2023 [50]	Good	Serious fall or fall‐related injury within 6–12 months after surgery	LTOT vs. short‐term opioid use and no use combined after surgery	AOR: 1.21 (1.05–1.39)
	Kim 2019 [41]	Good	Vertebral fracture within 90 days after surgery	LTOT vs. no opioid use before surgery	AHR: 2.47 (1.82–3.36)
			Non‐vertebral fracture within 90 days after surgery	LTOT vs. no opioid use before surgery	AHR: 1.49 (0.88–2.53)
Respiratory problem	Kim 2019 [41]	Good	Respiratory distress within 90 days after surgery	LTOT vs. no opioid use before surgery	AHR: 1.20 (0.52–2.72)
Bowel obstruction	Kim 2019 [41]	Good	Bowel obstruction within 90 days after surgery	LTOT vs. no opioid use before surgery	AHR: 1.79 (1.07–3.01)
Constipation	Won 2006 [57]	Fair	Constipation within 6 months	LTOT vs. no opioid use	AOR: 1.24 (0.87–1.76)
Surgical site infection	Ravi 2021 [48]	Good	Surgical site infection within 1 year after surgery	LTOT vs. no opioid use before surgery	AHR: 1.39 (1.02–1.90)
Traumatic brain injury	Herrera 2021 [36]	Good	Traumatic brain injury	LTOT vs. no opioid use/short‐term opioid use	AOR for LTOT vs. nonuse: 1.65 (1.49–1.82) AOR for LTOT vs. acute use: 0.93 (0.85–1.02)

Abbreviations: AHR, adjusted hazard ratio; AMR, adjusted mean ratio; AOR, adjusted odds ratio; ED, emergency department; LTOT, long‐term opioid therapy.

#### 3.3.1 Mortality

Two studies assessed the association between LTOT and mortality. One of them found that participants receiving LTOT after surgery had a greater hazard of mortality (unspecified cause) (Adjusted hazard ratio [AHR]: 3.44, 95% CI: 2.99–3.96) within 6 to 12 months after surgery compared to those who were either opioid‐naïve or received short‐term opioids [50]. The other study compared patients receiving preoperative LTOT with opioid‐naïve patients and found no difference in the hazard of all‐cause mortality within 90 days after surgery [41].

#### 3.3.2 Opioid Overdose

One study examined the association between LTOT and opioid overdose and found that participants receiving preoperative LTOT had a greater hazard of opioid overdose within 90 days (AHR: 9.60, 95% CI: 3.16–29.15) after surgery compared to opioid‐naïve participants [41].

#### 3.3.3 Health Care Utilization

Three studies investigated the relationship between preoperative LTOT and hospital readmission or ED visit after surgery, and two found positive associations [41, 48, 55]. One study found a positive association between postoperative LTOT and hospital readmission or ED visit [50]. Two studies found positive associations between preoperative LTOT and revision surgery [41, 48].

#### 3.3.4 Falls and Fractures

One study reported that participants receiving LTOT after surgery had greater odds of serious fall or fall‐related injury (Adjusted odds ratio [AOR]: 1.21, 95% CI: 1.05–1.39) within 6 to 12 months after surgery compared to those receiving short‐term opioids and not using opioids [50]. Another study investigated the risk of vertebral and nonvertebral fracture with preoperative LTOT versus no opioid use [41]. LTOT was positively associated with the hazard of vertebral fracture within 90 days (AHR: 2.47, 95% CI: 1.82–3.36) after surgery. However, no association was observed between LTOT and nonvertebral fracture.

#### 3.3.5 Other Adverse Outcomes

One study found that patients receiving LTOT after surgery had greater odds of hospital readmission/ED visit related to a respiratory event (AOR: 1.67, 95% CI: 1.49–1.86) within the 6 to 12 months after surgery compared to those receiving short‐term opioids and not using opioids [50]. However, another study did not find any significant difference in the 90‐days hazard of respiratory distress between LTOT patients and opioid‐naïve patients [41]. The same study also examined the association between preoperative LTOT and 90‐days hazard of postoperative bowel obstruction and observed a positive relationship (AHR: 1.79, 95% CI: 1.07–3.01) [41]. One study assessed the association between LTOT and constipation and did not observe any difference between LTOT and no opioid use (AOR: 1.24, 95% CI: 0.87–1.76) [57]. One study examined if LTOT in the year preceding surgery was associated with increased hazard of surgical site infection within 1 year after surgery and found a positive association (AHR: 1.39, 95% CI: 1.02–1.90) [48]. None of the studies included suicide, cardiac arrhythmia, or sleep apnea as outcomes.

#### 3.3.6 Economic Outcomes

Two studies investigated economic outcomes of LTOT after surgery and found that LTOT was associated with increased health care cost during the first postoperative year, with adjusted mean ratios ranging from 1.41 to 1.44 with LTOT [37, 39].

## 4 Discussion

This review is, to our best knowledge, the first systematic review to examine both the predictors and clinical outcomes associated with LTOT in older adults. In this review, the patient‐related factors associated with LTOT across most studies were low income/wealth, depressive disorders, and dual enrollment/eligibility in Medicare and Medicaid/Veterans Health Administration benefits. Notably, substance use disorders were not significantly associated with LTOT in most of the included studies, a finding that is in contrast with evidence from studies of the general population [64, 65]. In addition, we identified several characteristics of the initial prescription that predicted LTOT according to most studies including longer initial duration, long‐acting opioids, and prior/concurrent use of benzodiazepines, anxiolytics/sedatives/hypnotics, and anticonvulsants. Notably, many of these prescribing characteristics are also key areas of emphasis in the Centers for Disease Control and Prevention clinical practice guideline for prescribing opioids for pain [15]. It is important to note that patient‐related predictors are mostly non‐modifiable and are beyond the scope of a clinician's' intervention. On the other hand, prescription‐related predictors are modifiable, and clinicians can adopt prescribing strategies that are less likely to predispose patients to LTOT. Regarding the outcomes of LTOT, most studies reported positive associations between LTOT and hospital readmission/ED visit, revision surgery, opioid overdose, falls, and health care costs. Evidence on the association of LTOT with mortality and fractures was inconclusive.

Older adults are a heterogeneous population and factors such as frailty, comorbidities, and cognitive conditions may impact treatment outcomes. Based on our review, findings on the relationship between comorbidity score/number and LTOT were inconclusive and evidence was lacking for frailty. Also, dementia/Alzheimer's disease was not associated with LTOT. With regards to age, we did not observe any consistent pattern in its relationship with LTOT, suggesting all age groups above 60 years have similar risks of receiving LTOT. Another important factor that needs to be considered when prescribing opioids to older adults is whether they are using other psychotropic medications since polypharmacy is common among older adults. Most of the studies included in this review identified the use of benzodiazepines, anticonvulsants, and anxiolytics/sedatives/hypnotics as risk factors of LTOT. Therefore, prescribers should be cautious when prescribing opioids to patients who are on these medications. Regarding the clinical outcomes of LTOT, none of the included studies conducted subgroup analyses to determine whether the association between LTOT and clinical outcomes varied across patient groups defined by frailty, comorbidity number/score, age, dementia, or other cognitive conditions. This remains a critical evidence gap which future studies should address.

In most of the included studies assessing clinical outcomes of LTOT, LTOT was found to be associated with adverse outcomes such as hospital readmission/ED visit, revision surgery, opioid overdose, falls, and health care costs. However, we found no studies that assessed the effectiveness of LTOT in managing pain specifically among older adults. A comprehensive benefit‐harm assessment of LTOT is not possible without a clear understanding of its long‐term effectiveness in pain management. Even in the general population, the evidence regarding the effectiveness of LTOT remains inconclusive. A 2015 meta‐analysis found insufficient evidence to determine the effectiveness of LTOT for improving chronic pain, function, or quality of life [7]. A systematic review of open‐label extension trials identified some evidence supporting the effectiveness of LTOT in pain management; however, the quality of the evidence was rated as very poor quality [66]. More recent studies of the general population suggest that LTOT is no more effective than short‐term opioid therapy or nonopioid treatment in long‐term chronic pain management [67, 68, 69]. One of these studies reported that although approximately 20% of patients experienced significant benefit from LTOT, the majority did not [67]. Our findings indicate potential harms of LTOT and a lack of evidence supporting benefits of LTOT in older adults. It is important to note that opioids are often needed for effective pain control (particularly in patients undergoing surgery or trauma), and opioids are sometimes the only way to effectively manage acute pain. Our findings support efforts to carefully evaluate the ongoing needs for opioids (including benefits versus harms), and for those who are already on LTOT, consider deprescribing opioids when potential harms of LTOT outweigh the benefits [70].

This review has some limitations. First, due to large variations in the definition of LTOT used by studies, it was not possible to set a strict definition of LTOT as an inclusion criteria. Instead, we used a more flexible definition where 90 days of continuous opioid use, episodes of opioid use lasting 90 days, or any prescription after 90 days from the index date were considered to meet the criteria for LTOT. Similarly, there is no universal definition for “older adults” [71]. We chose a definition of ≥ 60 years old to facilitate a more comprehensive search (had we used a definition of ≥ 65 years old, only four studies would be excluded [45, 47, 51, 58], so it is unlikely this would significantly impact our findings). No randomized controlled studies meeting our criteria were identified, so the review relies on observational studies only. The studies included in this review had variations in the comparison group against the LTOT group. Studies compared long‐term opioid users with non‐users, short‐term opioid users, or both combined. And some studies did not specify the characteristics of the comparison group. These variations precluded meta‐analysis, and instead, we used harvest plots and narrative synthesis of the data to describe the predictors and clinical outcomes of LTOT. A strength of harvest plots is that they provide effective and easy‐to‐understand visual summaries of the distribution of evidence on a given hypothesis (positive, negative, or no association) from complex and heterogeneous studies. However, harvest plots are susceptible to subjective and potentially incorrect interpretation by readers since they do not provide any summary or pooled estimates of effect size or statistical significance. Although harvest plots show direction of association, no information on the magnitude of association can be derived from them. Additionally, there were evidence gaps among the included studies. As discussed previously, information on the effectiveness of LTOT was not available. Second, the majority of the studies did not provide any information on the dose of opioids being prescribed/dispensed. Seventeen of the 41 studies reported dosing information, and the time period for which the dose was measured varied among the studies (baseline, initial prescription, follow‐up, baseline and follow‐up). Most studies only used daily dose as a categorical variable and did not provide adequate summary measures of daily dose such as mean, median, or standard deviation.

## 5 Conclusions

Evidence from the included studies suggests that low income/wealth, depressive disorders, dual insurance eligibility/enrollment, prior/concurrent use of benzodiazepines, anxiolytics/sedatives/hypnotics, and anticonvulsants, long‐acting opioids, longer duration of initial opioids, and opioid use before or after surgery/trauma are associated with LTOT in older adults. Factors specifically relevant for older adults such as age, comorbidities, and dementia/Alzheimer's disease had mixed evidence, and evidence was lacking for frailty. The evidence also suggests that LTOT is associated with adverse outcomes such as hospital readmission/ED visit, revision surgery, opioid overdose, falls, and health care costs. However, none of the studies assessed the adverse outcomes of LTOT in subgroups defined by frailty, comorbidities, age, dementia, or other cognitive conditions. Moreover, no studies assessed the effectiveness of LTOT in improving pain specifically in older adults. To address this evidence gap, prospective studies with long‐term follow‐up comparing the effectiveness of LTOT with short‐term opioid therapy or long‐term therapy with nonopioid analgesics are needed.

## Author Contributions

**Iftekhar Ahmed:** conceptualization, methodology, software, investigation, formal analysis, writing – original draft, writing – review and editing. **Nina E. Teo:** conceptualization, methodology, investigation, writing – original draft, writing – review and editing, software. **Noha Keshk:** investigation, writing – review and editing, software. **David R. Foster:** conceptualization, methodology, supervision, writing – review and editing, software.

## Funding

The authors have nothing to report.

## Conflicts of Interest

The authors declare no conflicts of interest.

## Supporting information


**Table S1:** Amendments to the original protocol.
**Table S2:** Characteristics of the studies included in the review.
**Table S3:** Detailed characteristics of the included studies.

**Data S1:** Supporting Information.

## Data Availability

This systematic review is based on a review of published literature. All data used in this study are available in the main paper and its Supporting Information.

## References

[1] K. V. Patel , J. M. Guralnik , E. J. Dansie , and D. C. Turk , “Prevalence and Impact of Pain Among Older Adults in the United States: Findings From the 2011 National Health and Aging Trends Study,” Pain 154, no. 12 (2013): 2649–2657, 10.1016/j.pain.2013.07.029. 24287107 PMC3843850

[2] S. M. Rikard , “Chronic Pain Among Adults—United States, 2019–2021,” MMWR. Morbidity and Mortality Weekly Report 72 (2023): 379–385. 37053114 10.15585/mmwr.mm7215a1 PMC10121254

[3] M. C. Bicket , K.‐P. Chua , P. Lagisetty , et al., “Prevalence of Surgery Among Individuals in the United States,” Annals of Surgery Open 5, no. 2 (2024): e421. 38911632 10.1097/AS9.0000000000000421 PMC11191855

[4] American Geriatrics Society Panel on the Pharmacological Management of Persistent Pain in Older Person , “Pharmacological Management of Persistent Pain in Older Persons,” Pain Medicine 10, no. 6 (2009): 1062–1083. 19744205 10.1111/j.1526-4637.2009.00699.x

[5] B. E. Hoots , L. Xu , M. Kariisa , et al., “Annual Surveillance Report of Drug‐Related Risks and Outcomes—United States,” (2018).

[6] P. Schofield , M. Dunham , D. Martin , et al., “Evidence‐Based Clinical Practice Guidelines on the Management of Pain in Older People—A Summary Report,” British Journal of Pain 16, no. 1 (2022): 6–13. 35111309 10.1177/2049463720976155 PMC8801690

[7] R. Chou , J. A. Turner , E. B. Devine , et al., “The Effectiveness and Risks of Long‐Term Opioid Therapy for Chronic Pain: A Systematic Review for a National Institutes of Health Pathways to Prevention Workshop,” Annals of Internal Medicine 162, no. 4 (2015): 276–286. 25581257 10.7326/M14-2559

[8] M. Von Korff , A. Kolodny , R. A. Deyo , and R. Chou , Long‐Term Opioid Therapy Reconsidered (American College of Physicians, 2011), 325–328. 10.1059/0003-4819-155-5-201109060-00011 PMC3280085 21893626

[9] R. N. Karmali , A. C. Skinner , J. G. Trogdon , M. Weinberger , S. Z. George , and K. Hassmiller Lich , “The Association Between the Supply of Nonpharmacologic Providers, Use of Nonpharmacologic Pain Treatments, and High‐Risk Opioid Prescription Patterns Among Medicare Beneficiaries With Persistent Musculoskeletal Pain,” Medical Care 58, no. 5 (2020): 433–444, 10.1097/mlr.0000000000001299. 32028525 PMC7451631

[10] J. N. Hunnicutt , S. A. Chrysanthopoulou , C. M. Ulbricht , A. L. Hume , J. Tjia , and K. L. Lapane , “Prevalence of Long‐Term Opioid Use in Long‐Stay Nursing Home Residents,” Journal of the American Geriatrics Society 66, no. 1 (2018): 48–55, 10.1111/jgs.15080. 28940193 PMC5777877

[11] C. I. Campbell , C. Weisner , L. LeResche , et al., “Age and Gender Trends in Long‐Term Opioid Analgesic Use for Noncancer Pain,” American Journal of Public Health 100, no. 12 (2010): 2541–2547. 20724688 10.2105/AJPH.2009.180646 PMC2978198

[12] G. T. Ray , A. L. Bahorik , P. C. VanVeldhuisen , C. M. Weisner , A. L. Rubinstein , and C. I. Campbell , “Prescription Opioid Registry Protocol in an Integrated Health System,” American Journal of Managed Care 23, no. 5 (2017): e146. 28810131 PMC5560074

[13] J. M. Fritz , J. B. King , and C. McAdams‐Marx , “Associations Between Early Care Decisions and the Risk for Long‐Term Opioid Use for Patients With Low Back Pain With a New Physician Consultation and Initiation of Opioid Therapy,” Clinical Journal of Pain 34, no. 6 (2018): 552–558, 10.1097/ajp.0000000000000571. 29135698

[14] M. I. Bromley , E. P. Gain , M. A. Ray , F. Mzayek , S. K. Kedia , and X. Yu , “Burden of Chronic and Heavy Opioid Use Among Elderly Community Dwellers in the US,” AJPM Focus 3, no. 2 (2024): 100175. 38298247 10.1016/j.focus.2023.100175 PMC10828592

[15] D. Dowell , “CDC Clinical Practice Guideline for Prescribing Opioids for Pain—United States, 2022,” MMWR ‐ Recommendations and Reports 71 (2022): 1–95. 10.15585/mmwr.rr7103a1 PMC9639433 36327391

[16] M. J. Page , J. E. McKenzie , P. M. Bossuyt , et al., “The PRISMA 2020 Statement: An Updated Guideline for Reporting Systematic Reviews,” BMJ (Clinical Research Ed.) 372 (2021): n71, 10.1136/bmj.n71. PMC8005924 33782057

[17] J. de Oliveira Costa , C. Bruno , N. Baranwal , et al., “Variations in Long‐Term Opioid Therapy Definitions: A Systematic Review of Observational Studies Using Routinely Collected Data (2000–2019),” British Journal of Clinical Pharmacology 87, no. 10 (2021): 3706–3720, 10.1111/bcp.14798. 33629352

[18] R. N. Karmali , C. Bush , S. R. Raman , C. I. Campbell , A. C. Skinner , and A. W. Roberts , “Long‐Term Opioid Therapy Definitions and Predictors: A Systematic Review,” Pharmacoepidemiology and Drug Safety 29, no. 3 (2020): 252–269. 31851773 10.1002/pds.4929 PMC7058495

[19] M. Crowther , A. Avenell , G. MacLennan , and G. Mowatt , “A Further Use for the Harvest Plot: A Novel Method for the Presentation of Data Synthesis,” Research Synthesis Methods 2, no. 2 (2011): 79–83, 10.1002/jrsm.37. 26061676

[20] D. Ogilvie , D. Fayter , M. Petticrew , et al., “The Harvest Plot: A Method for Synthesising Evidence About the Differential Effects of Interventions,” BMC Medical Research Methodology 8, no. 1 (2008): 8, 10.1186/1471-2288-8-8. 18298827 PMC2270283

[21] G. A. Wells , B. Shea , D. O'Connell , et al., “The Newcastle‐Ottawa Scale (NOS) for Assessing the Quality of Nonrandomised Studies in Meta‐Analyses,” (2000), https://www.ohri.ca/programs/clinical_epidemiology/oxford.asp.

[22] M. A. Hillen , N. M. Medendorp , J. G. Daams , and E. M. A. Smets , “Patient‐Driven Second Opinions in Oncology: A Systematic Review,” Oncologist 22, no. 10 (2017): 1197–1211, 10.1634/theoncologist.2016-0429. 28606972 PMC5634767

[23] A. Alam , T. Gomes , H. Zheng , M. M. Mamdani , D. N. Juurlink , and C. M. Bell , “Long‐Term Analgesic Use After Low‐Risk Surgery: A Retrospective Cohort Study,” Archives of Internal Medicine 172, no. 5 (2012): 425–430, 10.1001/archinternmed.2011.1827. 22412106

[24] K. Beyene , H. Fahmy , A. H. Y. Chan , A. Tomlin , and G. Cheung , “Predictors of Persistent Opioid Use in Non‐Cancer Older Adults: A Retrospective Cohort Study,” Age and Ageing 52, no. 9 (2023): afad167, 10.1093/ageing/afad167. 37659093

[25] T. Bongiovanni , S. Gan , E. Finlayson , et al., “Prolonged Use of Newly Prescribed Gabapentin After Surgery,” Journal of the American Geriatrics Society 70, no. 12 (2022): 3560–3569, 10.1111/jgs.18005. 36000860 PMC9771946

[26] C. S. Brown , N. H. Osborne , H. M. Hu , et al., “Endovascular Surgery Is Not Protective Against New Persistent Opioid Use Development Compared to Open Vascular Surgery,” Vascular 30, no. 4 (2022): 728–738, 10.1177/17085381211024514. 34128428

[27] C. Chen , P. Tighe , W. H. Lo‐Ciganic , A. G. Winterstein , and Y. J. Jenny Wei , “Perioperative Use of Gabapentinoids and Risk for Postoperative Long‐Term Opioid Use in Older Adults Undergoing Total Knee or Hip Arthroplasty,” Journal of Arthroplasty 37 (2022): 2149–2157, 10.1016/j.arth.2022.05.018. 35577053 PMC9588599

[28] P. W. Chui , L. A. Bastian , E. DeRycke , C. A. Brandt , W. C. Becker , and J. L. Goulet , “Dual Use of Department of Veterans Affairs and Medicare Benefits on High‐Risk Opioid Prescriptions in Veterans Aged 65 Years and Older: Insights From the VA Musculoskeletal Disorders Cohort,” Health Services Research 53 (2018): 5402–5418, 10.1111/1475-6773.13060. 30298672 PMC6235820

[29] M. A. Cupp , F. L. Beaudoin , K. N. Hayes , et al., “Post‐Acute Care Setting After Hip Fracture Hospitalization and Subsequent Opioid Use in Older Adults,” Journal of the American Medical Directors Association 24, no. 7 (2023): 971–977, 10.1016/j.jamda.2023.03.012. 37080246 PMC10293035

[30] R. Daoust , J. Paquet , L. Moore , et al., “Incidence and Risk Factors of Long‐Term Opioid Use in Elderly Trauma Patients,” Annals of Surgery 268, no. 6 (2018): 985–991, 10.1097/sla.0000000000002461. 28767563

[31] L. D. Delaney , V. Gunaseelan , H. Rieck , J. M. Dupree , B. R. Hallstrom , and J. F. Waljee , “High‐Risk Prescribing Increases Rates of New Persistent Opioid Use in Total Hip Arthroplasty Patients,” Journal of Arthroplasty 35, no. 9 (2020): 2472–2479, 10.1016/j.arth.2020.04.019. 32389404 PMC8289485

[32] R. J. Desai , Y. Jin , P. D. Franklin , et al., “Association of Geography and Access to Health Care Providers With Long‐Term Prescription Opioid Use in Medicare Patients With Severe Osteoarthritis: A Cohort Study,” Arthritis and Rheumatology 71, no. 5 (2019): 712–721, 10.1002/art.40834. 30688044 PMC6483834

[33] C. Gopalakrishnan , R. J. Desai , J. M. Franklin , et al., “Development of a Medicare Claims‐Based Model to Predict Persistent High‐Dose Opioid Use After Total Knee Replacement,” Arthritis Care & Research (Hoboken) 74 (2022): 1342–1348, 10.1002/acr.24559. PMC8280246 33450136

[34] K. Hadlandsmyth , B. C. Lund , Y. Gao , et al., “Social Determinants of Long‐Term Opioid Use Following Total Knee Arthroplasty,” Journal of Knee Surgery 37 (2024): 742–748, 10.1055/s-0044-1786021. 38599604 PMC11542899

[35] T. E. Hereford , A. Porter, 3rd , J. B. Stambough , S. M. Cherney , and S. C. Mears , “Prevalence of Chronic Opioid Use in the Elderly After Hip Fracture Surgery,” Journal of Arthroplasty 37 (2022): S530–S535, 10.1016/j.arth.2022.01.071. 35219575

[36] A. V. Herrera , L. Wastila , J. P. Brown , H. Chen , S. R. Gambert , and J. S. Albrecht , “Effects of Prescription Opioid Use on Traumatic Brain Injury Risk in Older Adults,” Journal of Head Trauma Rehabilitation 36, no. 5 (2021): 388–395, 10.1097/htr.0000000000000716. 34489389 PMC8428555

[37] A. Johnson , B. Milne , N. Jamali , et al., “Chronic Opioid Use After Joint Replacement Surgery in Seniors Is Associated With Increased Healthcare Utilization and Costs: A Historical Cohort Study,” Canadian Journal of Anesthesia 69 (2022): 963–973, 10.1007/s12630-022-02240-1. 35314993

[38] A. Johnson , B. Milne , M. Pasquali , et al., “Long‐Term Opioid Use in Seniors Following Hip and Knee Arthroplasty in Ontario: A Historical Cohort Study,” Canadian Journal of Anesthesia 69 (2021): 934–944, 10.1007/s12630-021-02091-2. 34435322

[39] A. Johnson , F. Nguyen , M. Richardson , et al., “Postdischarge Opioid Use After Lumbar Spine Surgery Among Older Adults in Ontario: A Population‐Based Cohort Study,” Canadian Journal of Surgery 67, no. 3 (2024): E252–e260, 10.1503/cjs.003723. PMC11230664 38925858

[40] J. F. Karp , C. W. Lee , J. McGovern , G. Stoehr , C. C. Chang , and M. Ganguli , “Clinical and Demographic Covariates of Chronic Opioid and Non‐Opioid Analgesic Use in Rural‐Dwelling Older Adults: The MoVIES Project,” International Psychogeriatrics 25, no. 11 (2013): 1801–1810, 10.1017/s104161021300121x. 23883528 PMC4020176

[41] S. C. Kim , Y. Jin , Y. C. Lee , et al., “Association of Preoperative Opioid Use With Mortality and Short‐Term Safety Outcomes After Total Knee Replacement,” JAMA Network Open 2, no. 7 (2019): e198061, 10.1001/jamanetworkopen.2019.8061. 31365106 PMC6669774

[42] D. P. Ly , “Differences Within Practices in Opioid‐Prescribing Patterns of Orthopedic Surgeons and in Subsequent Rates of Chronic Opioid Use, 2012–2014,” Journal of General Internal Medicine 34, no. 4 (2019): 529–531, 10.1007/s11606-018-4745-7. 30604123 PMC6445834

[43] S. Musich , S. S. Wang , L. Slindee , S. Kraemer , and C. S. Yeh , “Characteristics Associated With Transition From Opioid Initiation to Chronic Opioid Use Among Opioid‐Naïve Older Adults,” Geriatric Nursing 40, no. 2 (2019): 190–196, 10.1016/j.gerinurse.2018.10.003. 30401575

[44] H. H. Nestvold , S. S. Skurtveit , A. Hamina , V. Hjellvik , and I. Odsbu , “Socioeconomic Risk Factors for Long‐Term Opioid Use: A National Registry‐Linkage Study,” European Journal of Pain (London, England) 28, no. 1 (2024): 95–104, 10.1002/ejp.2163. 37501355

[45] B. M. Nørgård , C. T. Thorarinsson , F. D. Zegers , et al., “The Use of Opioids Nine Months After Surgery for Crohn's Disease—A Nationwide Cohort Study,” Alimentary Pharmacology and Therapeutics 60, no. 1 (2024): 52–60, 10.1111/apt.18014. 38693747

[46] G. Oh , E. L. Abner , D. W. Fardo , P. R. Freeman , and D. C. Moga , “Patterns and Predictors of Chronic Opioid Use in Older Adults: A Retrospective Cohort Study,” PLoS One 14, no. 1 (2019): e0210341, 10.1371/journal.pone.0210341. 30633773 PMC6329525

[47] K. Okike , R. N. Chang , P. H. Chan , E. W. Paxton , and H. A. Prentice , “Prolonged Opioid Usage Following Hip Fracture Surgery in Opioid‐Naïve Older Patients,” Journal of Arthroplasty 38, no. 8 (2023): 1528–1534, 10.1016/j.arth.2023.01.069. 36773664

[48] B. Ravi , D. Pincus , R. Croxford , et al., “Patterns of Pre‐Operative Opioid Use Affect the Risk for Complications After Total Joint Replacement,” Scientific Reports 11, no. 1 (2021): 22124, 10.1038/s41598-021-01179-5. 34764305 PMC8586234

[49] K. B. Santosa , H. M. Hu , C. M. Brummett , et al., “New Persistent Opioid Use Among Older Patients Following Surgery: A Medicare Claims Analysis,” Surgery 167, no. 4 (2020): 732–742, 10.1016/j.surg.2019.04.016. 31349994 PMC7216555

[50] K. B. Santosa , C. R. Priest , J. D. Oliver , et al., “Long‐Term Health Outcomes of New Persistent Opioid Use After Surgery Among Medicare Beneficiaries,” Annals of Surgery 278, no. 3 (2023): E491–E495, 10.1097/SLA.0000000000005752. 36375090 PMC13052505

[51] J. P. Sardi , J. S. Smith , J. L. Gum , et al., “Opioid Use Prior to Adult Spine Deformity Correction Surgery Is Associated With Worse Pre‐ and Postoperative Back Pain and Prolonged Opioid Demands,” Global Spine Journal 15 (2024): 1749–1759, 10.1177/21925682241261662. 38832400 PMC11571721

[52] A. H. Simoni , L. Nikolajsen , A. E. Olesen , C. F. Christiansen , S. P. Johnsen , and A. B. Pedersen , “The Association Between Initial Opioid Type and Long‐Term Opioid Use After Hip Fracture Surgery in Elderly Opioid‐Naïve Patients,” Scandinavian Journal of Pain 20, no. 4 (2020): 755–764, 10.1515/sjpain-2019-0170. 32853173

[53] D. H. Solomon , J. Avorn , P. S. Wang , et al., “Prescription Opioid Use Among Older Adults With Arthritis or Low Back Pain,” Arthritis and Rheumatism 55, no. 1 (2006): 35–41, 10.1002/art.21697. 16463409

[54] J. M. Stone , A. Pujari , J. Garlich , and C. Lin , “A Retrospective Cohort Study on Chronic Opioid Use After Geriatric Hip Fracture Surgery‐Risk Factors, Trends, and Outcomes,” Journal of the American Academy of Orthopaedic Surgeons 31, no. 6 (2023): 312–318, 10.5435/jaaos-d-22-00458. 36595589

[55] R. Tang , K. B. Santosa , J. V. Vu , et al., “Preoperative Opioid Use and Readmissions Following Surgery,” Annals of Surgery 275, no. 1 (2022): E99–E106, 10.1097/SLA.0000000000003827. 32187028 PMC7935087

[56] K. Tevik , J. Benth , M. Aarøen , M. T. Lornstad , S. Bergh , and A. S. Helvik , “Prevalence and Persistent Use of Analgesic Drugs in Older Adults Receiving Domiciliary Care at Baseline‐A Longitudinal Study,” Health Science Reports 4, no. 3 (2021): e316, 10.1002/hsr2.316. 34250268 PMC8247935

[57] A. Won , K. L. Lapane , S. Vallow , J. Schein , J. N. Morris , and L. A. Lipsitz , “Long‐Term Effects of Analgesics in a Population of Elderly Nursing Home Residents With Persistent Nonmalignant Pain,” Journals of Gerontology. Series A, Biological Sciences and Medical Sciences 61, no. 2 (2006): 165–169, 10.1093/gerona/61.2.165. 16510860 PMC2276585

[58] A. K. Moffat , N. L. Pratt , M. Kerr , L. M. K. Ellett , and E. E. Roughead , “Risk of Chronic Opioid Use in Older Persons With Pre‐Existing Anxiety,” Journal of Opioid Management 16, no. 1 (2020): 59–66, 10.5055/jom.2020.0551. 32091618

[59] I. Ahmed , A. J. Zillich , N. L. Campbell , K. M. Sowinski , and D. R. Foster , “Long‐Term Opioid Therapy in Older Adults: Incidence and Risk Factors Related to Patient Characteristics and Initial Opioid Dispensed,” Journal of the American Pharmacists Association 65, no. 2 (2025): 102311, 10.1016/j.japh.2024.102311. 39667526

[60] N. Risbo , V. Ehrenstein , P. H. Gundtoft , J. E. Gjertsen , and A. B. Pedersen , “Socioeconomic Position and Chronic Opioid Use After Hip Fracture Surgery: A Danish Population‐Based Cohort Study,” European Journal of Pain 29, no. 6 (2025): e70063. 40536348 10.1002/ejp.70063 PMC12178151

[61] D. P. Ly , “Association of Patient Race and Ethnicity With Differences in Opioid Prescribing by Primary Care Physicians for Older Adults With New Low Back Pain,” JAMA Health Forum 2, no. 9 (2021): e212333, 10.1001/jamahealthforum.2021.2333. 35977182 PMC8796941

[62] S. R. Ytterberg , M. L. Mahowald , and S. R. Woods , “Codeine and Oxycodone Use in Patients With Chronic Rheumatic Disease Pain,” Arthritis & Rheumatism: Official Journal of the American College of Rheumatology 41, no. 9 (1998): 1603–1612. 10.1002/1529-0131(199809)41:9<1603::AID-ART10>3.0.CO;2-U 9751092

[63] J. R. Caldwell , R. J. Rapoport , J. C. Davis , et al., “Efficacy and Safety of a Once‐Daily Morphine Formulation in Chronic, Moderate‐To‐Severe Osteoarthritis Pain: Results From a Randomized, Placebo‐Controlled, Double‐Blind Trial and an Open‐Label Extension Trial,” Journal of Pain and Symptom Management 23, no. 4 (2002): 278–291, 10.1016/S0885-3924(02)00383-4. 11997197

[64] A. Mohamadi , J. J. Chan , J. Lian , et al., “Risk Factors and Pooled Rate of Prolonged Opioid Use Following Trauma or Surgery: A Systematic Review and Meta‐(Regression) Analysis,” Journal of Bone and Joint Surgery 100, no. 15 (2018): 1332–1340, 10.2106/jbjs.17.01239. 30063596

[65] J. Gong , P. Jones , and A. H. Y. Chan , “Incidence and Risk Factors of New Persistent Opioid Use After Surgery and Trauma: A Systematic Review,” BMC Surgery 24, no. 1 (2024): 210, 10.1186/s12893-024-02494-0. 39014357 PMC11251237

[66] P. Bialas , C. Maier , P. Klose , and W. Häuser , “Efficacy and Harms of Long‐Term Opioid Therapy in Chronic Non‐Cancer Pain: Systematic Review and Meta‐Analysis of Open‐Label Extension Trials With a Study Duration ≥ 26 Weeks,” European Journal of Pain 24, no. 2 (2020): 265–278, 10.1002/ejp.1496. 31661587

[67] H. Saïdi , P. M. Gabrielle , B. Aline , M. A. Ware , and M. Choinière , “Effectiveness of Long‐Term Opioid Therapy Among Chronic Non‐Cancer Pain Patients Attending Multidisciplinary Pain Treatment Clinics: A Quebec Pain Registry Study,” Canadian Journal of Pain 2, no. 1 (2018): 113–124, 10.1080/24740527.2018.1451252. 35005371 PMC8730575

[68] J. C. Licciardone , K. Rama , A. Nguyen , C. R. Prado , C. Stanteen , and S. Aryal , “Effectiveness of Long‐Term Opioid Therapy for Chronic Low Back Pain,” Journal of the American Board of Family Medicine 37, no. 1 (2024): 59–72, 10.3122/jabfm.2023.230140R1. 38092436

[69] E. E. Krebs , A. Gravely , S. Nugent , et al., “Effect of Opioid vs Nonopioid Medications on Pain‐Related Function in Patients With Chronic Back Pain or Hip or Knee Osteoarthritis Pain: The SPACE Randomized Clinical Trial,” Journal of the American Medical Association 319, no. 9 (2018): 872–882, 10.1001/jama.2018.0899. 29509867 PMC5885909

[70] C. Lin Chung‐Wei and V. Langford Aili , “Opioid Deprescribing in Patients With Noncancer Pain,” New England Journal of Medicine 393, no. 18 (2025): 1833–1842, 10.1056/NEJMcp2414789. 41191942

[71] A. M. Carew and C. Comiskey , “Treatment for Opioid Use and Outcomes in Older Adults: A Systematic Literature Review,” Drug and Alcohol Dependence 182 (2018): 48–57. 29136566 10.1016/j.drugalcdep.2017.10.007

