# Supplementary material for: Predictors and Clinical Outcomes of Long‐Term Opioid Therapy in Older Adults: A Systematic Review
Source: Pharmacotherapy. 2026 May 11;46:e70151. doi: 10.1002/phar.70151 (PMC13159056; doi:10.1002/phar.70151)
Supplement: Supplementary file 1 — Table S1: Amendments to the original protocol. Table S2: Characteristics of the studies included in the review. Table S3: Detailed characteristics of the included studies. [file PHAR-46-0-s002.pdf]

Predictors and clinical outcomes of long-term opioid therapy in older adults: A systematic review

Supplementary materials

Contents

Table S1: Amendments to the original protocol 2
Table S2: Characteristics of the studies included in the review. 3
Table S3: Detailed characteristics of the included studies 5
References 10

Table S1: Amendments to the original protocol

Amendment No.	Section	Details of amendments
1	Study inclusion criteria	Our protocol initially stated that case series studies would be included in the review. However, we later decided to exclude case series as well as case reports since they cannot be used to statistically assess relationship between the predictors and outcomes.
2	Databases for literature search	Our protocol initially stated that Medline and Cochrane databases would be searched. However, we decided to add a third database (Embase) to increase the comprehensiveness of our search.
3	Study variables	We added more details to the list of predictor variables, but the types/classes of variables remain the same. We also added economic outcomes as an outcome of interest.
4	Risk of bias assessment	Our protocol initially stated that NIH quality assessment tools would be used for risk of bias assessment. The authors later decided that the Newcastle-Ottawa Scales would be a better fit for the types of studies included in our review.

Table S2: Characteristics of the studies included in the review.

Characteristics	No. studies (N=41)
**Year of publication**	
2006-2010	2
2011-2015	2
2016-2020	13
2021-2025	24
**Study location**	
United States	27
Canada	6
Denmark	3
Norway	2
Australia	1
New Zealand	1
Multiple countries	1
**Research question**	
Predictors of LTOT	30
Outcomes of LTOT	8
Both predictors and outcomes of LTOT	3
**Study design**	
Retrospective cohort studies	34
Cross-sectional studies	3
Prospective cohort studies	2
Case-control studies	1
Case-crossover studies	1
**Pain conditions studied**	
Surgery/trauma	27
Chronic pain	6
Chronic pain and surgery/trauma	3
Not mentioned	5
**Minimum age of older adults for inclusion**	
>/≥65 years	30
>/≥66 years	4

>/≥60 years	4
>/≥67 years	1
>/≥68 years	1
>/≥70 years	1
**Definition of LTOT**	
Duration ≥90 days	11
Last prescription/dispensation after ≥90 days from the index date	6
Duration ≥180 days	5
Based on trajectory modelling	4
Last prescription/dispensation after ≥180 days from the index date	3
Multiple criteria	3
Other criteria	10
**Comparison group against LTOT users**	
Short-term opioid users	19
Opioid non-users	7
Both short-term users and non-users	4
Participants serving as their own control	1
Unclear	10

LTOT= Long-term opioid therapy

Table S3: Detailed characteristics of the included studies

Study ID	Study location	Research question	Study design	Conditions studied	Minimum age of older adults for inclusion	Definition of LTOT	Comparison group	Sample size
Ahmed 2025 [1]	United States	Predictors of LTOT	Retrospective cohort study	Chronic pain & surgery/trauma	Age ≥65 years	Opioid use episode > 90 days and >60 cumulative days' supply	Received short-term opioid therapy	162,287
Alam 2012 [2]	Canada	Predictors of LTOT	Retrospective cohort study	Surgery/trauma	Age ≥66 years	Receipt of opioids between 305-425 days after the index date (the date of hospital discharge)	Did not receive any opioid	391,139
Beyene 2023 [3]	New Zealand	Predictors of LTOT	Retrospective cohort study	Chronic pain	Age ≥65 years	Continuously filling ≥1 opioid prescription within 91-180 days after the index opioid use (1st opioid dispensing date during the study period)	Received short-term opioid therapy	268,857
Bongiovanni 2022 [4]	United States	Outcomes of LTOT	Retrospective cohort study	Surgery/trauma	Age ≥66 years	Opioid prescription refilled between 90-180 days after discharge from surgery	Unclear	17,481
Brown 2022 [5]	United States	Predictors of LTOT	Retrospective cohort study	Surgery/trauma	Age ≥65 years	One or more opioid prescription fills both between 4-90 and 91-180 days postoperatively	Unclear	77,767
Chen 2022 [6]	United States	Predictors of LTOT	Retrospective cohort study	Surgery/trauma	Age ≥65 years	Opioid duration ≥ 90 days	Received short-term opioid therapy	52,788
Chui 2018 [7]	United States	Predictors of LTOT	Cross sectional study	Chronic pain	Age ≥65 years	Opioid duration > 90 days and overlap of concurrent opioid prescriptions in 2010	Received short-term opioid therapy	21,111
Cupp 2023 [8]	United States	Predictors of LTOT	Retrospective cohort study	Surgery/trauma	Age ≥66 years	An episode of 90 or more days of continuous use with gaps of no more than 30 days in the supply of opioid dispensed	Unclear	101,021
Daoust 2018 [9]	Canada	Predictors of LTOT	Retrospective cohort study	Surgery/trauma	Age ≥65 years	Filling at least 1 opioid prescription from 305 to 425 days after the hospital discharge	Did not receive any opioid	38,963
Delaney 2020 [10]	United States	Predictors of LTOT	Retrospective cohort study	Surgery/trauma	Age ≥65 years	Filling an opioid prescription during the perioperative period, as well as	Unclear	1,403

Study ID	Study location	Research question	Study design	Conditions studied	Minimum age of older adults for inclusion	Definition of LTOT	Comparison group	Sample size
						an additional fill from 91 to 180 days after discharge from surgery		
Desai 2019 [11]	United States	Predictors of LTOT	Retrospective cohort study	Surgery/trauma	Age ≥65 years	Dispensing for any opioid with the day supply totaling ≥90 days in a 360-day period	Unclear	358,121
Gopalakrishnan 2021 [12]	United States	Predictors of LTOT	Retrospective cohort study	Surgery/trauma	Age ≥65 years	Trajectory models were used to identify LTOT	Received short-term opioid therapy	142,089
Hadland smyth 2024 [13]	United States	Predictors of LTOT	Retrospective cohort study	Surgery/trauma	Age ≥65 years	>90 days of opioid use beginning within 90 days post surgery	Received short-term opioid therapy	9,064
Hereford 2022 [14]	United States	Predictors of LTOT	Retrospective cohort study	Surgery/trauma	Age ≥65 years	Filling an opioid prescription between 6 months and 1 year post surgery	Received short-term opioid therapy	219
Herrera 2021 [15]	United States	Outcomes of LTOT	Case crossover study	Surgery/trauma	Age ≥65 years	Opioid use during the entirety of any 90-day exposure interval	Patients served as their own controls	50,873
Hunnicutt 2018 [16]	United States	Predictors of LTOT	Cross sectional study	Not mentioned	Age ≥65 years	≥90 days cumulative use of opioid during the 120-day study period	Received short term and medium-term opioid therapy + did not receive any opioid	315,949
Johnson 2021 [17]	Canada	Predictors of LTOT	Retrospective cohort study	Surgery/trauma	Age >65 years	The last opioid prescription was filled between 181–365 days after surgical discharge	Received short-term opioid therapy	135,196
Johnson 2022 [18]	Canada	Outcomes of LTOT	Retrospective cohort study	Surgery/trauma	Age >65 years	The last opioid prescription was filled between 181–365 days after surgical discharge	Received short-term opioid therapy	135,196
Johnson 2024 [19]	Canada	Both predictors and outcomes of LTOT	Retrospective cohort study	Surgery/trauma	Age ≥65 years	No clear definition of LTOT provided. However, in logistic regression, the event was opioid use>271 days.	Received short-term opioid therapy	15,109

Study ID	Study location	Research question	Study design	Conditions studied	Minimum age of older adults for inclusion	Definition of LTOT	Comparison group	Sample size
Karmali 2020 [20]	United States	Predictors of LTOT	Retrospective cohort study	Chronic pain	Age >65 years	Days' supply > 90 days	Received short-term opioid therapy	65,101
Karp 2013 [21]	United States	Predictors of LTOT	Prospective cohort study	Not mentioned	Age ≥65 years	Trajectory models were used to identify LTOT	Both infrequent and non-users of opioids	1,109
Kim 2019 [22]	United States	Outcomes of LTOT	Retrospective cohort study	Surgery/trauma	Age ≥65 years	≥1 opioid dispensing in each of the past 12 months before surgery	Did not receive any opioid	316,593; subgroup analysis of 256,553 patients with no malignant tumors was done
Ly 2019 [23]	United States	Predictors of LTOT	Retrospective cohort study	Surgery/trauma	Age ≥65 years	180 days or more of opioids supplied in the 365 days subsequent to the procedure, excluding the first 30 days	Received short-term opioid therapy	2,691
Ly 2021[24]	United States	Predictors of LTOT	Cross sectional study	Chronic pain	Age ≥66 years	Being prescribed at least 180 days of opioids in days 366 to 730 (the year after the new low back pain episode).	Unclear	274,771
Moffat 2020 [25]	Australia	Predictors of LTOT	Retrospective cohort study	Not mentioned	Age ≥60 years	Dispensing of sufficient opioid supply for 90 continuous days of treatment	Received short-term opioid therapy	15,000
Musich 2019 [26]	United States	Predictors of LTOT	Retrospective cohort study	Chronic pain & surgery/trauma	Age ≥65 years	≥2 prescriptions and > 90 days' supply of opioids	Received short-term opioid therapy	180,498
Nestvold 2024 [27]	Norway	Predictors of LTOT	Case control study	Not mentioned	Age ≥68 years	(1) had a first dispensation and then a second within 91–180 days from the first one and (2) were	Received short-term opioid therapy	Total: 1078,210; 68

Study ID	Study location	Research question	Study design	Conditions studied	Minimum age of older adults for inclusion	Definition of LTOT	Comparison group	Sample size
						dispensed ≥90 administration units (e.g., tablets) of opioids within the first 90 days		years and above: 431,685
Nørgård 2024 [28]	Denmark	Predictors of LTOT	Retrospective cohort study	Surgery/trauma	Age ≥60 years	≥1 filled prescription in at least two of three consecutive quarters after index surgery	Unclear	Total: 5,186 Older adults: 797
Oh 2019 [29]	United States	Predictors of LTOT	Retrospective cohort study	Chronic pain	Age ≥65 years	Trajectory models were used to identify LTOT	Received short-term opioid therapy	13,059
Okike 2023 [30]	United States	Predictors of LTOT	Retrospective cohort study	Surgery/trauma	Age ≥60 years	Filling ≥1 opioid prescriptions in all three time periods (day 0 to 30, day 31 to 90, day 91 to 180)	Did not receive any opioid	47,309
Ravi 2021 [31]	Canada	Outcomes of LTOT	Retrospective cohort study	Surgery/trauma	Age ≥67 years	≥90 days of continuous use of opioids	Did not receive any opioid	110,130
Risbo 2025 [32]	Denmark	Predictors of LTOT	Retrospective cohort study	Surgery/trauma	Age ≥65 years	≥2 opioid prescriptions for 92–365 days after surgery	Unclear	52,801
Santosa 2020 [33]	United States	Predictors of LTOT	Retrospective cohort study	Surgery/trauma	Age ≥65 years	Opioid prescription filled between 90 and 180 days after surgery	Received short-term opioid therapy	81,839
Santosa 2023 [34]	United States	Outcomes of LTOT	Retrospective cohort study	Surgery/trauma	Age ≥65 years	A prescription opioid fill both in the first 90-day period after surgery (excluding the perioperative period of 30 d before to 3 d after surgery) and 91 to 180 days period after surgery	Received short term opioid therapy + did not receive any opioid	229,898
Sardi 2024 [35]	Multiple countries	Both predictors and outcomes of LTOT	Retrospective cohort study	Surgery/trauma	Age ≥60 years	Continued opioid use at 2 years postoperatively	Unclear	219
Simoni 2020 [36]	Denmark	Predictors of LTOT	Retrospective cohort study	Surgery/trauma	Age ≥65 years	≥1 redeemed prescription within each of the 3 3-month periods (3-6	Received short-term opioid therapy	21,255

Study ID	Study location	Research question	Study design	Conditions studied	Minimum age of older adults for inclusion	Definition of LTOT	Comparison group	Sample size
						months, 6-9 months, and 9-12 months after hip fracture surgery) within the year after hip fracture surgery		
Solomon 2006 [37]	United States	Predictors of LTOT	Retrospective cohort study	Chronic pain	Age ≥65 years	At least a 180-day continuous supply of opioids.	Unclear	18,099
Stone 2023 [38]	United States	Both predictors and outcomes of LTOT	Retrospective cohort study	Surgery/trauma	Age >65 years	Uninterrupted opioid use with continued refills through 6 months postoperatively	Received short term opioid therapy + did not receive any opioid	322
Tang 2022 [39]	United States	Outcomes of LTOT	Retrospective cohort study	Surgery/trauma	Age ≥65 years	A machine learning algorithm was used to identify LTOT	Did not receive opioid	373,991
Tevik 2021 [40]	Norway	Predictors of LTOT	Prospective cohort study	Not mentioned	Age ≥70 years	Opioid use at 18 months and 36 months after the baseline data collection period	Received short-term opioid therapy	1,001
Won 2006 [41]	United States	Outcomes of LTOT	Retrospective cohort study	Chronic pain & surgery/trauma	Age ≥65 years	Opioid use for ≥ 6 months	Did not receive any opioid	3,669

LTOT= Long-term opioid therapy

References

1. Ahmed I, Zillich AJ, Campbell NL, Sowinski KM, Foster DR. Long-term opioid therapy in older adults: Incidence and risk factors related to patient characteristics and initial opioid dispensed. *Journal of the American Pharmacists Association*. 2025/03/01/ 2025;65(2):102311. doi:https://doi.org/10.1016/j.japh.2024.102311
2. Alam A, Gomes T, Zheng H, Mamdani MM, Juurlink DN, Bell CM. Long-term analgesic use after low-risk surgery: a retrospective cohort study. *Arch Intern Med*. 2012;172(5):425-30. doi:10.1001/archinternmed.2011.1827
3. Beyene K, Fahmy H, Chan AHY, Tomlin A, Cheung G. Predictors of persistent opioid use in non-cancer older adults: a retrospective cohort study. *Age and Ageing*. 2023;52(9)doi:10.1093/ageing/afad167
4. Bongiovanni T, Gan S, Finlayson E, et al. Prolonged use of newly prescribed gabapentin after surgery. *Journal of the American Geriatrics Society*. 2022;70(12):3560-3569. doi:10.1111/jgs.18005
5. Brown CS, Osborne NH, Hu HM, et al. Endovascular surgery is not protective against new persistent opioid use development compared to open vascular surgery. *Vascular*. 2022;30(4):728-738. doi:10.1177/17085381211024514
6. Chen C, Tighe P, Lo-Ciganic WH, Winterstein AG, Jenny Wei YJ. Perioperative Use of Gabapentinoids and Risk for Postoperative Long-term Opioid Use in Older Adults Undergoing Total Knee or Hip Arthroplasty. *J Arthroplasty*. 2022;doi:10.1016/j.arth.2022.05.018
7. Chui PW, Bastian LA, DeRycke E, Brandt CA, Becker WC, Goulet JL. Dual Use of Department of Veterans Affairs and Medicare Benefits on High-Risk Opioid Prescriptions in Veterans Aged 65 Years and Older: Insights from the VA Musculoskeletal Disorders Cohort. *Health Services Research*. 2018;53:5402-5418. doi:10.1111/1475-6773.13060
8. Cupp MA, Beaudoin FL, Hayes KN, et al. Post-Acute Care Setting After Hip Fracture Hospitalization and Subsequent Opioid Use in Older Adults. *Journal of the American Medical Directors Association*. 2023;24(7):971-977.e4. doi:10.1016/j.jamda.2023.03.012
9. Daoust R, Paquet J, Moore L, et al. Incidence and Risk Factors of Long-term Opioid Use in Elderly Trauma Patients. *Ann Surg*. 2018;268(6):985-991. doi:10.1097/sla.0000000000002461
10. Delaney LD, Gunaseelan V, Rieck H, Dupree JM, Hallstrom BR, Waljee JF. High-Risk Prescribing Increases Rates of New Persistent Opioid Use in Total Hip Arthroplasty Patients. *Journal of Arthroplasty*. 2020;35(9):2472-2479.e2. doi:10.1016/j.arth.2020.04.019
11. Desai RJ, Jin Y, Franklin PD, et al. Association of Geography and Access to Health Care Providers With Long-Term Prescription Opioid Use in Medicare Patients With Severe Osteoarthritis: A Cohort Study. *Arthritis and Rheumatology*. 2019;71(5):712-721. doi:10.1002/art.40834
12. Gopalakrishnan C, Desai RJ, Franklin JM, et al. Development of a Medicare Claims-Based Model to Predict Persistent High-Dose Opioid Use After Total Knee Replacement. *Arthritis Care Res (Hoboken)*. 2021;doi:10.1002/acr.24559
13. Hadlandsmyth K, Lund BC, Gao Y, et al. Social Determinants of Long-Term Opioid Use Following Total Knee Arthroplasty. *Journal of Knee Surgery*. 2024;doi:10.1055/s-0044-1786021
14. Hereford TE, Porter A, 3rd, Stambough JB, Cherney SM, Mears SC. Prevalence of Chronic Opioid Use in the Elderly After Hip Fracture Surgery. *J Arthroplasty*. 2022;doi:10.1016/j.arth.2022.01.071
15. Herrera AV, Wastila L, Brown JP, Chen H, Gambert SR, Albrecht JS. Effects of Prescription Opioid Use on Traumatic Brain Injury Risk in Older Adults. *J Head Trauma Rehabil*. 2021;36(5):388-395. doi:10.1097/htr.0000000000000716

16. Hunnicutt JN, Chrysanthopoulou SA, Ulbricht CM, Hume AL, Tjia J, Lapane KL. Prevalence of Long-Term Opioid Use in Long-Stay Nursing Home Residents. *J Am Geriatr Soc*. 2018;66(1):48-55. doi:10.1111/jgs.15080
17. Johnson A, Milne B, Pasquali M, et al. Long-term opioid use in seniors following hip and knee arthroplasty in Ontario: a historical cohort study. *Can J Anaesth*. 2021;doi:10.1007/s12630-021-02091-2
18. Johnson A, Milne B, Jamali N, et al. Chronic opioid use after joint replacement surgery in seniors is associated with increased healthcare utilization and costs: a historical cohort study. *Canadian Journal of Anesthesia*. 2022;doi:10.1007/s12630-022-02240-1
19. Johnson A, Nguyen F, Richardson M, et al. Postdischarge opioid use after lumbar spine surgery among older adults in Ontario: a population-based cohort study. *Can J Surg*. 2024;67(3):E252-e260. doi:10.1503/cjs.003723
20. Karmali RN, Skinner AC, Trogdon JG, Weinberger M, George SZ, Hassmiller Lich K. The Association Between the Supply of Nonpharmacologic Providers, Use of Nonpharmacologic Pain Treatments, and High-risk Opioid Prescription Patterns Among Medicare Beneficiaries With Persistent Musculoskeletal Pain. *Med Care*. 2020;58(5):433-444. doi:10.1097/mlr.0000000000001299
21. Karp JF, Lee CW, McGovern J, Stoehr G, Chang CC, Ganguli M. Clinical and demographic covariates of chronic opioid and non-opioid analgesic use in rural-dwelling older adults: the MoVIES project. *Int Psychogeriatr*. 2013;25(11):1801-10. doi:10.1017/s104161021300121x
22. Kim SC, Jin Y, Lee YC, et al. Association of Preoperative Opioid Use With Mortality and Short-term Safety Outcomes After Total Knee Replacement. *JAMA Netw Open*. 2019;2(7):e198061. doi:10.1001/jamanetworkopen.2019.8061
23. Ly DP. Differences Within Practices in Opioid-Prescribing Patterns of Orthopedic Surgeons and in Subsequent Rates of Chronic Opioid Use, 2012–2014. *Journal of General Internal Medicine*. 2019;34(4):529-531. doi:10.1007/s11606-018-4745-7
24. Ly DP. Association of Patient Race and Ethnicity With Differences in Opioid Prescribing by Primary Care Physicians for Older Adults With New Low Back Pain. *JAMA Health Forum*. 2021;2(9):e212333. doi:10.1001/jamahealthforum.2021.2333
25. Moffat AK, Pratt NL, Kerr M, Ellett LMK, Roughead EE. Risk of chronic opioid use in older persons with pre-existing anxiety. *Journal of Opioid Management*. 2020;16(1):59-66. doi:10.5055/jom.2020.0551
26. Musich S, Wang SS, Slindee L, Kraemer S, Yeh CS. Characteristics associated with transition from opioid initiation to chronic opioid use among opioid-naïve older adults. *Geriatr Nurs*. 2019;40(2):190-196. doi:10.1016/j.gerinurse.2018.10.003
27. Nestvold HH, Skurtveit SS, Hamina A, Hjellvik V, Odsbu I. Socioeconomic risk factors for long-term opioid use: A national registry-linkage study. *European Journal of Pain (United Kingdom)*. 2024;28(1):95-104. doi:10.1002/ejp.2163
28. Nørgård BM, Thorarinsson CT, Zegers FD, et al. The use of opioids nine months after surgery for Crohn's disease – a nationwide cohort study. *Alimentary Pharmacology and Therapeutics*. 2024;60(1):52-60. doi:10.1111/apt.18014
29. Oh G, Abner EL, Fardo DW, Freeman PR, Moga DC. Patterns and predictors of chronic opioid use in older adults: A retrospective cohort study. *PLoS One*. 2019;14(1):e0210341. doi:10.1371/journal.pone.0210341
30. Okike K, Chang RN, Chan PH, Paxton EW, Prentice HA. Prolonged Opioid Usage Following Hip Fracture Surgery in Opioid-Naïve Older Patients. *Journal of Arthroplasty*. 2023;38(8):1528-1534.e1. doi:10.1016/j.arth.2023.01.069
31. Ravi B, Pincus D, Croxford R, et al. Patterns of pre-operative opioid use affect the risk for complications after total joint replacement. *Sci Rep*. 2021;11(1):22124. doi:10.1038/s41598-021-01179-5

32. Risbo N, Ehrenstein V, Gundtoft PH, Gjertsen JE, Pedersen AB. Socioeconomic Position and Chronic Opioid Use After Hip Fracture Surgery: A Danish Population-Based Cohort Study. *European Journal of Pain*. 2025;29(6):e70063.
33. Santosa KB, Hu HM, Brummett CM, et al. New persistent opioid use among older patients following surgery: A Medicare claims analysis. *Surgery*. 2020;167(4):732-742. doi:10.1016/j.surg.2019.04.016
34. Santosa KB, Priest CR, Oliver JD, et al. Long-term Health Outcomes of New Persistent Opioid Use after Surgery among Medicare Beneficiaries. *Annals of Surgery*. 2023;278(3):E491-E495. doi:10.1097/SLA.0000000000005752
35. Sardi JP, Smith JS, Gum JL, et al. Opioid Use Prior to Adult Spine Deformity Correction Surgery is Associated With Worse Pre- and Postoperative Back Pain and Prolonged Opioid Demands. *Global Spine Journal*. 2024;doi:10.1177/21925682241261662
36. Simoni AH, Nikolajsen L, Olesen AE, Christiansen CF, Johnsen SP, Pedersen AB. The association between initial opioid type and long-term opioid use after hip fracture surgery in elderly opioid-naïve patients. *Scand J Pain*. 2020;20(4):755-764. doi:10.1515/sjpain-2019-0170
37. Solomon DH, Avorn J, Wang PS, et al. Prescription opioid use among older adults with arthritis or low back pain. *Arthritis Rheum*. 2006;55(1):35-41. doi:10.1002/art.21697
38. Stone JM, Pujari A, Garlich J, Lin C. A Retrospective Cohort Study on Chronic Opioid Use After Geriatric Hip Fracture Surgery-Risk Factors, Trends, and Outcomes. *J Am Acad Orthop Surg*. 2023;31(6):312-318. doi:10.5435/jaaos-d-22-00458
39. Tang R, Santosa KB, Vu JV, et al. Preoperative Opioid Use and Readmissions following Surgery. *Annals of Surgery*. 2022;275(1):E99-E106. doi:10.1097/SLA.0000000000003827
40. Tevik K, Benth J, Aarøen M, Lornstad MT, Bergh S, Helvik AS. Prevalence and persistent use of analgesic drugs in older adults receiving domiciliary care at baseline-A longitudinal study. *Health Sci Rep*. 2021;4(3):e316. doi:10.1002/hsr2.316
41. Won A, Lapane KL, Vallow S, Schein J, Morris JN, Lipsitz LA. Long-term effects of analgesics in a population of elderly nursing home residents with persistent nonmalignant pain. *J Gerontol A Biol Sci Med Sci*. 2006;61(2):165-9. doi:10.1093/gerona/61.2.165
